# The potassium channel subunit K_V_1.8 (*Kcna10*) is essential for the distinctive outwardly rectifying conductances of type I and II vestibular hair cells

**DOI:** 10.7554/eLife.94342

**Published:** 2024-12-03

**Authors:** Hannah R Martin, Anna Lysakowski, Ruth Anne Eatock

**Affiliations:** 1 Department of Neurobiology, University of Chicago Chicago United States; 2 Department of Anatomy and Cell Biology, University of Illinois at Chicago Chicago United States; University of Oxford United Kingdom; University of Oxford United Kingdom

**Keywords:** hair cell, potassium channel, vestibular, inner ear, utricle, voltage gated, Mouse

## Abstract

In amniotes, head motions and tilt are detected by two types of vestibular hair cells (HCs) with strikingly different morphology and physiology. Mature type I HCs express a large and very unusual potassium conductance, g_K,L_, which activates negative to resting potential, confers very negative resting potentials and low input resistances, and enhances an unusual non-quantal transmission from type I cells onto their calyceal afferent terminals. Following clues pointing to K_V_1.8 (*Kcna10*) in the Shaker K channel family as a candidate g_K,L_ subunit, we compared whole-cell voltage-dependent currents from utricular HCs of K_V_1.8-null mice and littermate controls. We found that K_V_1.8 is necessary not just for g_K,L_ but also for fast-inactivating and delayed rectifier currents in type II HCs, which activate positive to resting potential. The distinct properties of the three K_V_1.8-dependent conductances may reflect different mixing with other K_V_ subunits that are reported to be differentially expressed in type I and II HCs. In K_V_1.8-null HCs of both types, residual outwardly rectifying conductances include K_V_7 (*Knq*) channels. Current clamp records show that in both HC types, K_V_1.8-dependent conductances increase the speed and damping of voltage responses. Features that speed up vestibular receptor potentials and non-quantal afferent transmission may have helped stabilize locomotion as tetrapods moved from water to land.

## Introduction

The receptor potentials of hair cells (HCs) are strongly shaped by large outwardly rectifying K^+^ conductances that are differentially expressed according to HC type. Here, we report that a specific voltage-gated K^+^ (K_V_) channel subunit participates in very different K_V_ channels dominating the membrane conductances of type I and II HCs in amniote vestibular organs.

Type I HCs occur only in amniote vestibular organs. Their most distinctive features are that they are enveloped by a calyceal afferent terminal (Wersall, 1956; Lysakowski and Goldberg, 2004) and that they express g_K,L_ (Correia and Lang, 1990; Rennie and Correia, 1994; Rüsch and Eatock, 1996a): a large non-inactivating conductance with an activation range from –100 to –60 mV, far more negative than other ‘low-voltage-activated’ K_V_ channels. HCs are known for their large outwardly rectifying K^+^ conductances, which repolarize membrane voltage following a mechanically evoked perturbation and in some cases contribute to sharp electrical tuning of the HC membrane. g_K,L_ is unusually large and unusually negatively activated, and therefore strongly attenuates and speeds up the receptor potentials of type I HCs (Correia et al., 1996; Rüsch and Eatock, 1996b). In addition, g_K,L_ augments non-quantal transmission from type I HC to afferent calyx by providing open channels for K^+^ flow into the synaptic cleft (Contini et al., 2012; Contini et al., 2017; Contini et al., 2020; Govindaraju et al., 2023), increasing the speed and linearity of the transmitted signal (Songer and Eatock, 2013).

Type II HCs have compact afferent synaptic contacts (boutons) where the receptor potential drives quantal release of glutamate. They have fast-inactivating (A-type, g_A_) and delayed rectifier (g_DR_) conductances that are opened by depolarization above resting potential (*V*_rest_).

The unusual properties of g_K,L_ have long attracted curiosity about its molecular nature. g_K,L_ has been proposed to include ‘M-like’ K_V_ channels in the K_V_7 and/or erg channel families (Kharkovets et al., 2000; Hurley et al., 2006; Holt et al., 2007). The K_V_7.4 subunit was of particular interest because it contributes to the low-voltage-activated conductance, g_K,n_, in cochlear outer HCs, but was eventually eliminated as a g_K,L_ subunit by experiments on K_V_7.4-null mice (Spitzmaul et al., 2013).

Several observations suggested the K_V_1.8 (KCNA10) subunit as an alternative candidate for g_K,L_. K_V_1.8 is highly expressed in vestibular sensory epithelia (Carlisle et al., 2012), particularly HCs (Lee et al., 2013; Scheffer et al., 2015; McInturff et al., 2018), with slight expression elsewhere (skeletal muscle, Lee et al., 2013; kidney, Yao et al., 2002). *Kcna10*^–/–^ mice show absent or delayed vestibular-evoked potentials, the synchronized activity of afferent nerve fibers sensitive to fast linear head motions (Lee et al., 2013). Unique among K_V_1 channels, K_V_1.8 has a cyclic nucleotide-binding domain (Lang et al., 2000) with the potential to explain g_K,L_’s known cGMP dependence (Behrend et al., 1997; Chen and Eatock, 2000).

Our comparison of whole-cell currents and immunohistochemistry in type I HCs from *Kcna10*^–/–^ and *Kcna10*^+/+,+/–^ mouse utricles showed that K_V_1.8 expression is necessary for g_K,L_. More surprisingly, K_V_1.8 expression is also required for A-type and delayed rectifier conductances of type II HCs. In both HC types, eliminating the K_V_1.8-dependent major conductances revealed a smaller delayed rectifier conductance involving K_V_7 channels. Thus, the distinctive outward rectifiers that produce such different receptor potentials in type I and II HCs both include K_V_1.8 and K_V_7 channels.

## Results

We compared whole-cell voltage-activated K^+^ currents in type I and II HCs from homozygous knockout (*Kcna10*^–/–^) animals and their wildtype (*Kcna10*^+/+^) or heterozygote (*Kcna10*^+/–^) littermates. We immunolocalized K_V_1.8 subunits in the utricular epithelium and pharmacologically characterized the residual K^+^ currents of *Kcna10*^–/–^ animals. Current clamp experiments demonstrated the impact of K_V_1.8-dependent currents on passive membrane properties.

We recorded from three utricular zones: lateral extrastriola (LES), striola, and medial extrastriola (MES); striolar and extrastriolar zones have many structural and functional differences and give rise to afferents with different physiology (reviewed in Goldberg, 2000; Eatock and Songer, 2011). Recordings are from 412 type I and II HCs (53% LES, 30% MES, 17% striola) from mice between postnatal day (P) 5 and P370. We recorded from such a wide age range to test for developmental or senescent changes in the impact of the null mutation. Above P18, we did not see substantial changes in K_V_ channel properties, as reported (González-Garrido et al., 2021).

*Kcna10*^–/–^ animals appeared to be healthy and to develop and age normally, as reported (Lee et al., 2013), and HCs were healthy (see Methods for criteria).

### K_V_1.8 is necessary for g_K,L_ in type I HCs

**Figure 1.**
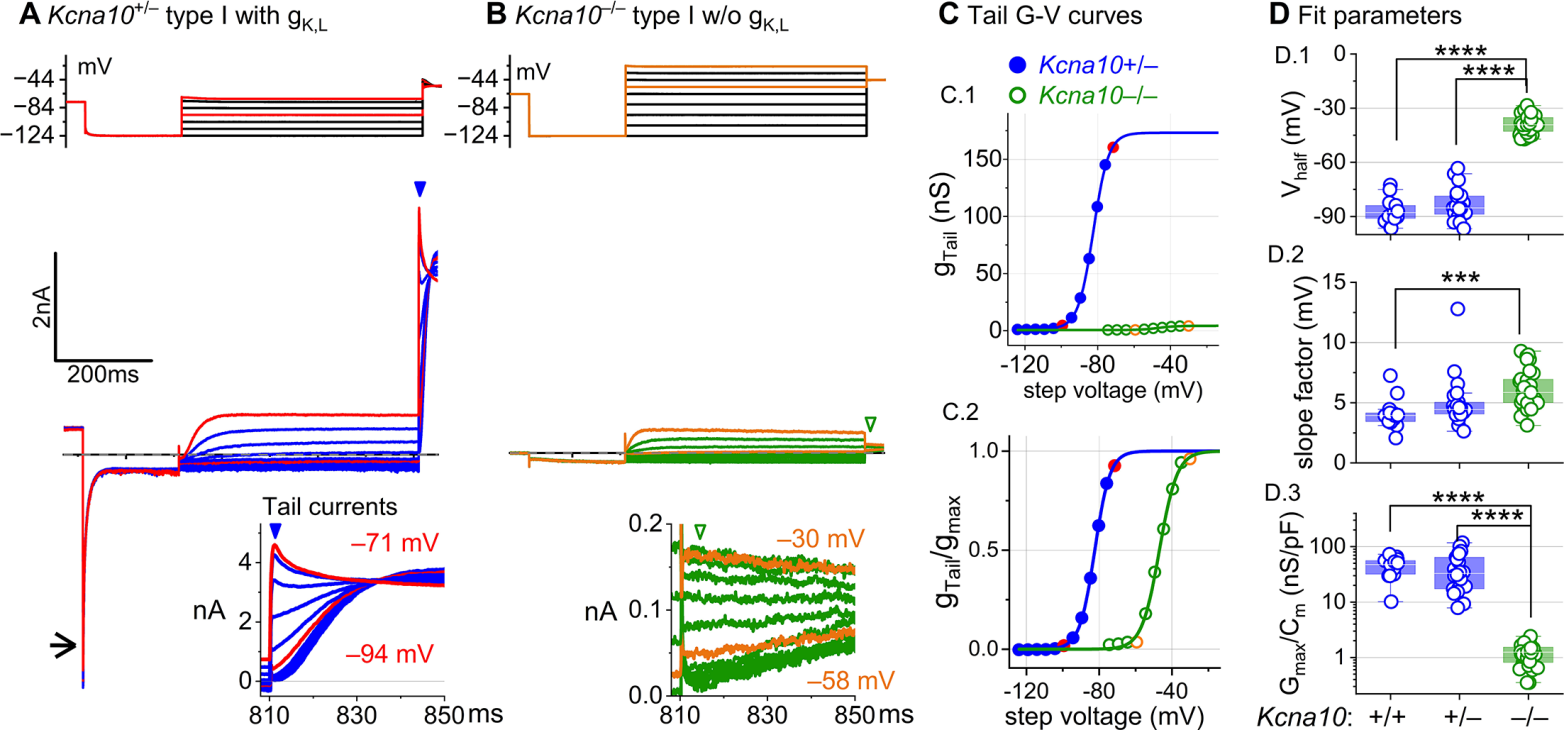
*Kcna10*^–/–^ type I hair cells (HCs) lacked g_K,L_, the dominant conductance in mature *Kcna10*^+/+,++/––^ type I HCs. Representative voltage-evoked currents in (**A**) a P22 *Kcna10*^+/–^ type I HC and (**B**) a P29 *Kcna10*^–/–^ type I HC. (**A**) *Arrow,* transient inward current that is a hallmark of g_K,L_. *Arrowheads,* tail currents, magnified in *insets*. For steps positive to the midpoint voltage, tail currents are very large. As a result, K^+^ accumulation in the calyceal cleft reduces driving force on K^+^, causing currents to decay rapidly, as seen in A (Lim et al., 2011). Note that the voltage protocol (top) in B extends to more positive voltages. (**C**) Activation (*G*–*V*) curves from tail currents in A and B; symbols, data; curves, Boltzmann fits (Equation 1). (**D**) Fit parameters from mice >P12 show big effect of *Kcna10*^–/–^ and no difference between *Kcna10*^+/–^ and *Kcna10*^+/+^. (**D.1**), Tukey’s test: +/+ vs –/–, p<1E-9; +/– vs –/–, p<1E-9. (**D.2**), Tukey’s test: +/+ vs –/–, p=9.4E-4. (**D.3**), Tukey’s test: +/+ vs –/–, p<1E-9; +/– vs –/–, p<1E-9. *Asterisks*: ***p < 0.001; and ****p < 0.0001. *Line,* median; *Box,* interquartile range; *Whiskers*, outliers. See Table 1 for statistics.

The large low-voltage-activated conductance, g_K,L_, in *Kcna10*^+/+,+/–^ type I HCs produces distinctive whole-cell current responses to voltage steps, as highlighted by our standard type I voltage protocol (Figure 1A). From a holding potential within the g_K,L_ activation range (here –74 mV), the cell is hyperpolarized to –124 mV, negative to *E*_K_ and the activation range, producing a large inward current through open g_K,L_ channels that rapidly decays as the channels deactivate. We use the large transient inward current as a hallmark of g_K,L_. The hyperpolarization closes all g_K,L_ channels, and then the activation function is probed with a series of depolarizing steps, obtaining the maximum conductance from the peak tail current at –44 mV (Figure 1A). We detected no difference between the Boltzmann parameters of g_K,L_ *G*–*V* curves from *Kcna10*^+/–^ and *Kcna10*^+/+^ type I HCs.

**Figure 1—figure supplement 1.**
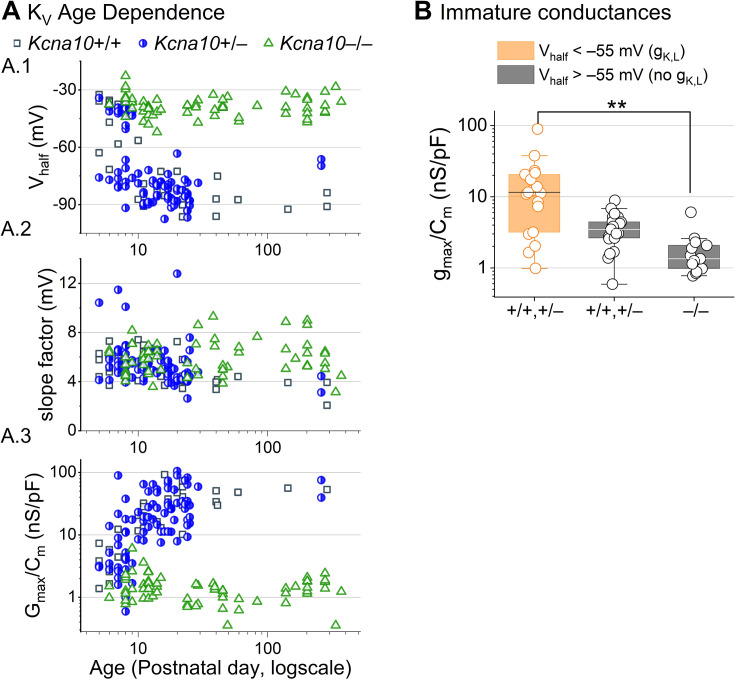
Developmental changes in type I hair cell (HC) K_V_ conductances. (**A**) Parameters from Boltzmann fits of tail *G*–*V* relations for type I HCs plotted against age. (**B**) Conductance density is similar in young (P5–P10) type I HCs that lack g_K,L_. g_K,L_ is defined here as having a *V*_half_ negative to –55 mV. *Kcna10*^+/+,+/–^ *with* g_K,L_, 17 ± 5 nS/pF (19); *Kcna10*^+/+,+/–^ *without* g_K,L_, 3.7 ± 0.4 nS/pF (22); *Kcna10*^–/–^, 1.8 ± 0.4 nS/pF (13). *Kcna10*^+/+,+/–^ *with* g_K,L_ vs *Kcna10*^–/–^: p = 0.007, KWA, g 1.0. *Asterisks:* **p < 0.01. *Line,* median; *Box,* interquartile range; *Whiskers*, outliers.

**Table 1.** Type I hair cell K_V_ activation voltage dependence. Mean ± SEM (number of cells). g is effect size, Hedge’s g. KWA is Kruskal–Wallis ANOVA.

Zone	Kcna10	Tail *V*_1/2_, mV*	Tail *S*, mV^†^	Tail g_max_, nS^‡^	Tail g_max_/*C*_m_, nS/pF^§^	Age (median, range)
Extrastriola	+/+	–85 ± 2 (12)	4.3 ± 0.4 (12)	270 ± 40 (11)	47 ± 8 (11)	22, 14–287
	+/–	–83 ± 1 (40)	5.2 ± 0.3 (40)	210 ± 20 (40)	37 ± 4 (40)	19, 13–259
	–/–	–40.2 ± 0.9 (26)	5.7 ± 0.3 (26)	5.4 ± 0.3 (26)	1.11 ± 0.08 (26)	45, 14–277
Striola	+/+	–87 ± 3 (6)	4.3 ± 0.3 (6)	310 ± 70 (6)	41 ± 7 (6)	40, 15–59
	+/–	–88 ± 2 (3)	4.7 ± 0.9 (3)	270 ± 60 (3)	44 ± 6 (3)	19, 14–20
	–/–	–38 ± 1 (13)	6.2 ± 0.4 (13)	6.5 ± 0.6 (13)	1.5 ± 0.1 (13)	202, 14–370

* –/– vs +/+: two-way ANOVA, p < 1E−9, g 7.7; –/– vs +/–: two-way ANOVA, p < 1E−9, g 6.8.

† –/– vs +/+: two-way ANOVA, p = 8.4E−4, g 1.2.

‡ –/– vs +/+: two-way ANOVA, p < 1E−9, g 3.7; –/– vs +/–: two-way ANOVA, p < 1E−9, g 2.1.

§ –/– vs +/+: two-way ANOVA, p < 1E−9, g 3.6; –/– vs +/–: two-way ANOVA, p < 1E−9, g 2.0.

For a similar voltage protocol, *Kcna10*^–/–^ type I HCs (Figure 1B) produced no inward transient current at the step from holding potential to –124 mV and much smaller depolarization-activated currents during the iterated steps, even at much more positive potentials. Figure 1C compares the conductance–voltage (*G*–*V*, activation) curves fit to tail currents (Equation 1; see insets in Figure 1A, B): the maximal conductance (g_max_) of the *Kcna10*^–/–^ HC was over 10-fold smaller (Figure 1C.1), and the curve was positively shifted by >40 mV (Figure 1C.2). Figure 1D shows the *G*–*V* Boltzmann fit parameters for type I HCs from mice >P12, an age at which type I HCs normally express g_K,L_ (Rüsch et al., 1998).

In type I HCs from *Kcna10*^+/+,+,–^ mice, the *G*–*V* parameters of outwardly rectifying currents transitioned over the first 15–20 postnatal days from values for a conventional delayed rectifier, activating positive to resting potential, to g_K,L_ values (Figure 1—figure supplement 1A), as previously described (Rüsch et al., 1998; Géléoc et al., 2004; Hurley et al., 2006). Between P5 and P10, some type I HCs have not yet acquired the physiologically defined conductance, g_K,L_. No effects of K_V_1.8 deletion were seen in the delayed rectifier currents of immature type I HCs (Figure 1—figure supplement 1B), showing that they were not immature forms of the K_V_1.8-dependent g_K,L_ channels.

*Kcna10*^–/–^ type I HCs had a much smaller residual delayed rectifier that activated positive to resting potential, with *V*_half_ ~–40 mV and g_max_ density of 1.3 nS/pF. No additional K^+^ conductance activated up to +40 mV, and *G*–*V* parameters did not change much with age from P5 to P370. We characterize this K_V_1.8-independent delayed rectifier later. A much larger non-g_K,L_ delayed rectifier conductance (g_DR,I_) was reported in our earlier publication on mouse utricular type I HCs (Rüsch et al., 1998). This current was identified as that remaining after ‘blocking’ g_K,L_ with 20 mM external Ba^2+^. Our new data suggest that there is no large non-g_K,L_ conductance, and that instead high Ba^2+^ positively shifted the apparent voltage dependence of g_K,L_.

### K_V_1.8 strongly affects type I passive properties and responses to current steps

While the cells of *Kcna10*^–/–^ and *Kcna10*^+/–^ epithelia appeared healthy, type I HCs had smaller membrane capacitances (*C*_m_): 4–5 pF in *Kcna10*^–/–^ type I HCs, ~20% smaller than *Kcna10*^+/–^ type I HCs (~6 pF) and ~30% smaller than *Kcna10*^+/+^ type I HCs (6–7 pF; Table 2). While *C*_m_ scales with surface area, the lack of change in soma sizes by deletion of K_V_1.8 (Supplementary file 1b) suggests that surface area was not different. Instead, *C* may be higher in *Kcna10*^+/+^ cells because of g_K,L_ for two reasons. First, highly expressed trans-membrane proteins (see discussion of g_K,L_ channel density in Chen and Eatock, 2000) can affect membrane thickness (Mitra et al., 2004), which is inversely proportional to specific *C*_m_. Second, resistive current through g_K,L_ could contaminate estimations of capacitive current, which is calculated from the decay time constant of transient current evoked by a small voltage step negative to –90 mV, where we measured *C*_m_ (see Methods).

**Table 2.** Type I hair cell passive membrane properties in the extrastriola (ES) and striola (S). Mean ± SEM (number of cells). g is effect size, Hedge’s g. KWA is Kruskal–Wallis ANOVA.

Zone	*Kcna10*	*V*_rest_, mV^*, †^	*R*_input_, MΩ^‡^	τ10RC, ms^_§_^	*C*_m_, pF^¶^	Age (median, range)
ES	+/+	–84 ± 3 (6)	44 ± 6 (6)	0.24 ± 0.03 (6)	6.1 ± 0.4 (13)	20, 14–287
	+/–	–88.0 ± 0.7 (28)	55 ± 5 (24)	0.32 ± 0.03 (23)	5.8 ± 0.2 (44)	21, 16–29
	–/–	–63 ± 2 (15)	1400 ± 100 (15)	6.4 ± 0.6 (15)	5.0 ± 0.2 (27)	45, 14–202
S	+/+	–87 ± 2 (4)	50 ± 8 (4)	0.30 ± 0.04 (4)	7.4 ± 0.7 (7)	43, 40–59
	+/–	–87 ± 3 (3)	38 ± 8 (2)	0.21 ± 0.01 (2)	5.9 ± 0.6 (3)	19, 19–20
	–/–	–74 ± 5 (5)	1000 ± 300 (4)	4.2 ± 1.0 (4)	4.4 ± 0.2 (14)	202, 24–370

* Striolar –/– vs ES –/–: two-way ANOVA, p = 0.006, g 1.2; striolar –/– vs striolar +/+,+/–: two-way ANOVA, p = 0.005, g 1.7.

† –/– vs +/+: two-way ANOVA, p < 1E−9, g 2.3; –/– vs +/–: two-way ANOVA, p < 1E−9, g 3.4.

‡ –/– vs +/+: two-way ANOVA, p < 1E−9, g 3.1; –/– vs +/–: two-way ANOVA, p < 1E−9, g 3.9.

§† –/– vs +/+: two-way ANOVA, p < 1E−9, g 2.7; –/– vs +/–: two-way ANOVA, p < 1E−9, g 3.4.

¶‡ –/– vs +/+: two-way ANOVA, p = 3E−7, g 1.5; –/– vs +/–: two-way ANOVA, p = 1.3E−4, g 1.0; +/–vs +/+: two-way ANOVA, p = 0.048, g 0.6.

**Figure 2.**
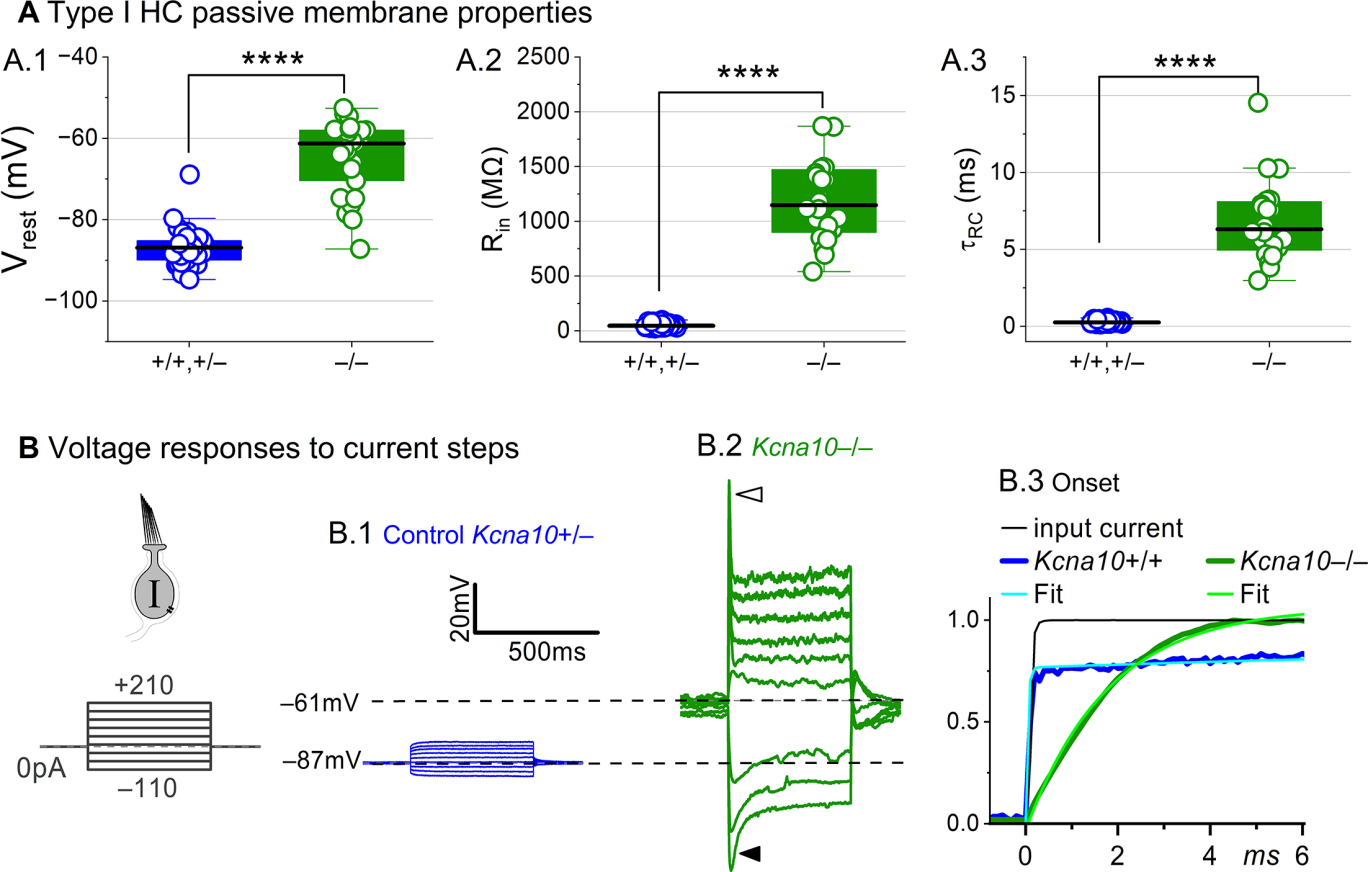
*Kcna10*^–/–^ type I hair cells (HCs) had much longer membrane charging times and higher input resistances (voltage gains) than *Kcna10*^+/+,+/–^ type I HCs. (**A**) g_K,L_ strongly affects passive membrane properties: (**A.1**) *V*_rest_, Tukey’s test p<1E-9, (**A.2**) *R*_in_, input resistance, Tukey’s test p<1E-9, and (**A.3**) membrane time constant, $\tau_{RC}=(R_{input}*C_{m})$, Tukey’s test p<1E-9. See Table 2 for all statistics. (**B**) Current clamp responses to the same scale from (**B.1**) *Kcna10*^+/–^ and (**B.2**) *Kcna10*^–/–^ type I cells, both P29. *Filled arrowhead (B.2),* sag indicating *I*_H_ activation. *Open arrowhead*, Depolarization rapidly decays as *I*_DR_ activates. (**B.3**) First 6 ms of voltage responses to 170 pA injection, normalized to steady-state value; *curves*, double-exponential fits (*Kcna10*^+/+^, τ 40 μs and 2.4 ms) and single-exponential fits (*Kcna10*^–/–^, τ 1.1 ms). *Asterisks,* ****p < 0.0001. *Line,* median; *Box,* interquartile range; *Whiskers*, outliers.

Basolateral conductances help set the resting potential and passive membrane properties that regulate the time course and gain of voltage responses to small currents. To examine the effect of K_V_1.8 on these properties, we switched to current clamp mode and measured resting potential (*V*_rest_), input resistance (*R*_in_, equivalent to voltage gain for small current steps, Δ*V*/Δ*I*), and membrane time constant ($\tau_{RC}$). In *Kcna10*^–/–^ type I HCs, *V*_rest_ was much less negative (Figure 2A.1), *R*_in_ was greater by ~20-fold (Figure 2A.2), and membrane charging times were commensurately longer (Figure 2A.3).

The differences between the voltage responses of *Kcna10*^+/+,+/–^ and *Kcna10*^–/–^ type I HCs are expected from the known impact of g_K,L_ on *V*_rest_ and *R*_in_ (Correia and Lang, 1990; Ricci et al., 1996; Rüsch and Eatock, 1996b; Songer and Eatock, 2013). The large K^+^-selective conductance at *V*_rest_ holds *V*_rest_ close to *E*_K_ (K^+^ equilibrium potential) and minimizes gain (Δ*V*/Δ*I*), such that voltage-gated conductances are negligibly affected by the input current and the cell produces approximately linear, static responses to iterated current steps. For *Kcna10*^–/–^ type I HCs, with their less negative *V*_rest_ and larger *R*_in_, positive current steps evoked a fast initial depolarization (Figure 2B.2), activating residual delayed rectifiers and repolarizing the membrane toward *E*_K_. Negative current steps evoked an initial hyperpolarization followed by a slowly repolarizing ‘sag’ (Figure 2B.2) as the HCN1 channels open (Rüsch and Eatock, 1996b; Horwitz et al., 2011).

Overall, comparison of the *Kcna10*^+/+,+/–^ and *Kcna10*^–/–^ responses shows that with K_V_1.8 (g_K,L_), the voltage response of the type I HC is smaller but better reproduces the time course of the input current.

### K_V_1.8 is necessary for both inactivating and non-inactivating K_V_ currents in type II HCs

Type II HCs also express K_V_1.8 mRNA (McInturff et al., 2018; Orvis et al., 2021). Although their outwardly rectifying conductances (g_A_ and g_DR_) differ substantially in voltage dependence and size from g_K,L_, both conductances were strongly affected by the null mutation: g_A_ was eliminated and the delayed rectifier was substantially smaller. Below we describe g_A_ and g_DR_ in *Kcna10*^+/+,+/–^ type II HCs and the residual outward-rectifying current in *Kcna10*^–/–^ type II HCs.

***Kcna10***^***+/+,+/–***^ ***type II HCs***. Most (81/84) *Kcna10*^+/+,+/–^ type II HCs expressed a rapidly activating, rapidly inactivating A-type conductance (g_A_). We define A current as the outwardly rectifying current that inactivates by over 30% within 200 ms. g_A_ was more prominent in extrastriolar zones, as reported (Holt et al., 1999; Weng and Correia, 1999).

**Figure 3.**
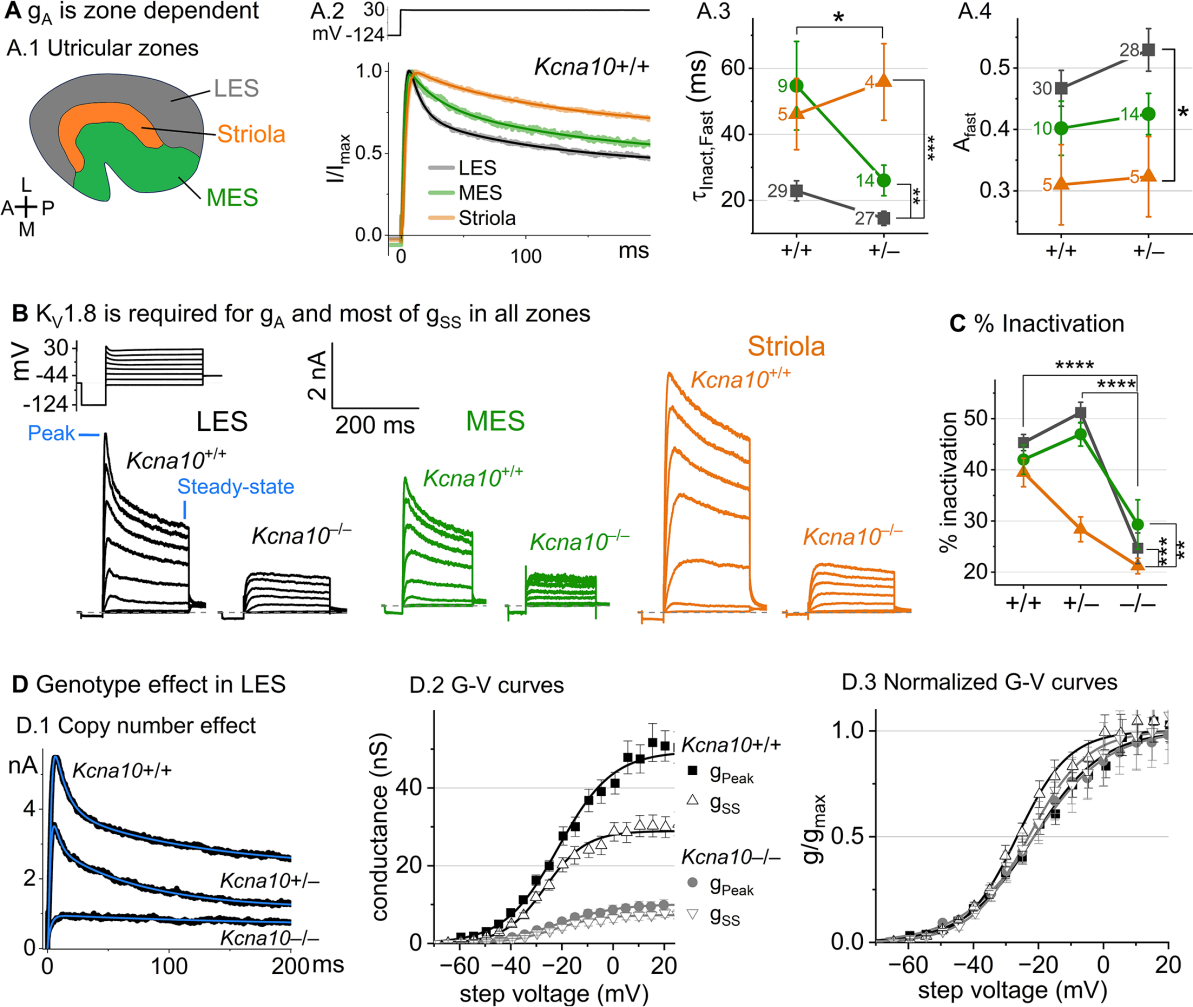
*Kcna10*^–/–^ type II hair cells (HCs) in all zones of the sensory epithelium lacked the major rapidly inactivating conductance, g_A_, and had less delayed rectifier conductance. Activation and inactivation varied with epithelial zone and genotype. (**A**) g_A_ inactivation time course varied across zones. (**A.1**) Zones of the utricular epithelium: lateral extrastriola (LES), medial extrastriola (MES), and striola (S). (**A.2**) Normalized currents evoked by steps from –124 to +30 mV with overlaid fits of Equation 3. (**A.3**) $\tau_{Inact,Fast}$ was faster in *Kcna10*^+/–^ (n=45) than *Kcna10*^+/+^ (n=43) HCs (KWA, p=0.027), and faster in LES (n=56) than MES (n=23, KWA, p=0.002) or S (n=9, KWA, p=2E-4). Point label is number of cells. Brackets show post hoc pairwise comparisons between two zones (vertical brackets) and horizontal brackets compare two genotypes; see Table 3 for statistics on kinetics. (**A.4**) Fast inactivation was a greater fraction of total inactivation in LES (n=58) than striola (n=10, Tukey’s test p=0.0041). (**B**) Exemplars; ages, *left to right*, P312, P53, P287, P49, P40, P154. (**C**) % inactivation at 30 mV was much lower in *Kcna10*^–/–^ (n=37) than *Kcna10*^+/–^ (n=47, Tukey’s HSD, p<1E-9) and *Kcna10*^+/+^ (n=44, Tukey’s HSD, p<1E-9). % inactivation was lower in striola (n=16) than LES (n=77, Tukey’s HSD, p=3E-5) and MES (n=36, Tukey’s HSD, p=0.0011). 2-way ANOVA detected interaction between zone and genotype, p=0.026 (Table 3). (**D**) Exemplar currents and *G*–*V* curves from LES type II HCs show a copy number effect. (**D.1**) Exemplar currents evoked by steps from –124 to +30 mV fit with Equation 3. (**D.2**) Averaged peak and steady-state conductance–voltage data points from LES cells (+/+, *n*=37; –/–, *n*=20) were fit with Boltzmann equations (Equation 1) and normalized by g_max_ in (**D.3**). *Asterisks*: *p < 0.05; **p < 0.01; ***p < 0.001; and ****p < 0.0001. *Error bars,* SEM. See Table 4 for statistics on voltage dependence.

We compared the activation and inactivation time course and inactivation prominence for 200 ms steps from –124 to ~30 mV. Outward currents fit with Equation 3 yielded fast inactivation time constants ($\tau_{Inact,Fast}$) of ~30 ms in LES (Figure 3A.2). $\tau_{Inact,Fast}$ was faster in LES than in MES or striola (Figure 3A.3) and fast inactivation was a larger fraction of the total inactivation in LES than striola (~0.5 vs 0.3, Figure 3A.4).

**Figure 3—figure supplement 1.**
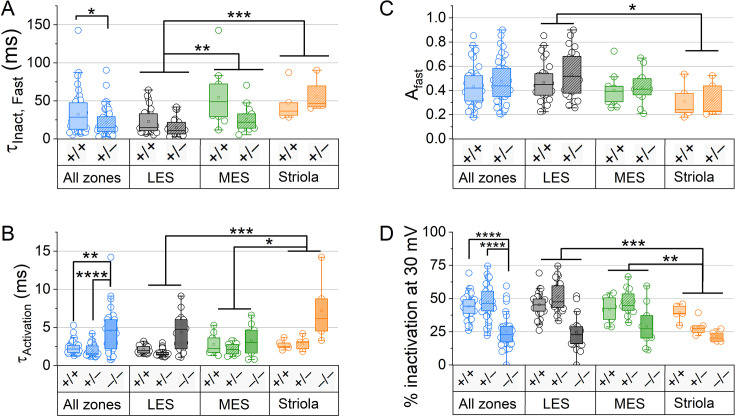
For type II hair cells (HCs) older than P12, K_V_ conductance activation and inactivation differed across zones and genotypes. (**A**) In *Kcna10^+/+^* and *Kcna10^+/–^* HCs, $\tau_{Inact,Fast}$ at 30 mV was faster in LES (n=56) than MES (n=23, KWA p=0.002) or S (n=9, KWA p=2E-4). $\tau_{Inact,Fast}$ was faster in *Kcna10*^+/–^ (n=45) than *Kcna10*^+/+^ (n=43, KWA p=0.027, see Table 3). (**B**) Fast inactivation was a larger fraction of total inactivation in LES (n=56) than striola (n=9, Tukey’s p=0.0041). (**C**) $\tau_{Act}$ at 30 mV was slower in *Kcna10*^–/–^ (n=53) than *Kcna10*^+/+^ (n=49, KWA p=0.0048) and *Kcna10*^+/–^ (n=59, KWA p=2E-7), and slower in S (n=16) than LES (n=75, KWA p=6E-4) and MES (n=33, KWA p=0.02). (**D**) Percent inactivation at 30 mV was lower in S (n=20) than LES (n=99, 2-way ANOVA Tukey’s p<0.0001) and MES (n=42, 2-way ANOVA Tukey’s p=0.001), and lower in *Kcna10*^–/–^ (n=43) than *Kcna10*^+/+^ (n=58, 2-way ANOVA Tukey’s p=0.0048 and <0.0001) and *Kcna10*^+/–^ (n=60, 2-way ANOVA Tukey’s p<0.0001). Interaction between Zone and Genotype was significant (p=0.026). *Asterisks*: *p < 0.05; **p < 0.01; ***p < 0.001; and ****p < 0.0001. *Line,* median; *Box,* interquartile range; *Whiskers*, outliers.

**Figure 3—figure supplement 2.**
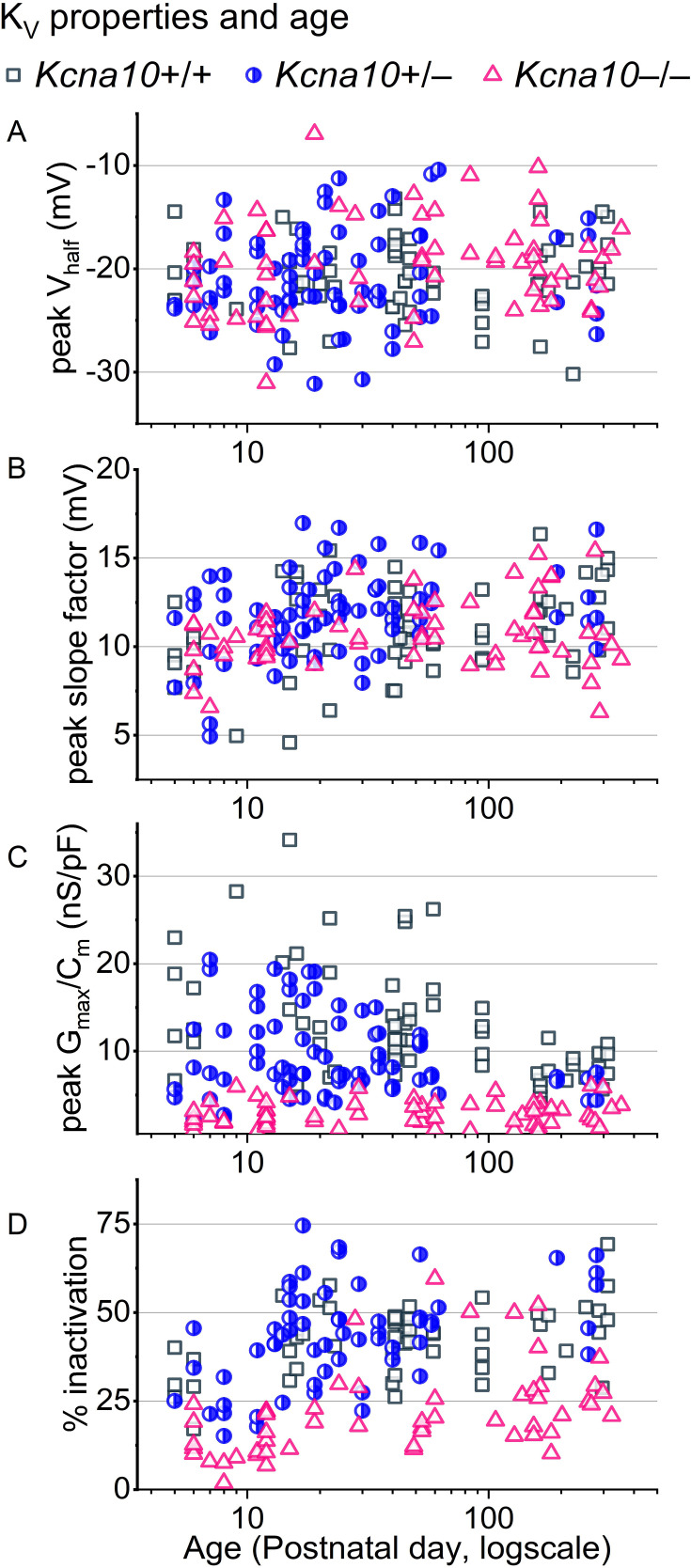
For type II hair cells (HCs) older than P12, K_V_ conductances were stable. (**A–C**) Parameters from Boltzmann fits of peak *G*–*V* relations and (**D**) % inactivation at +30 mV plotted against age from all zones. Overlaid curves are smoothing cubic β-splines. Note the seven extrastriolar Kcna10^–/–^ type II HCs with % inactivation >30%.

**Figure 3—figure supplement 3.**
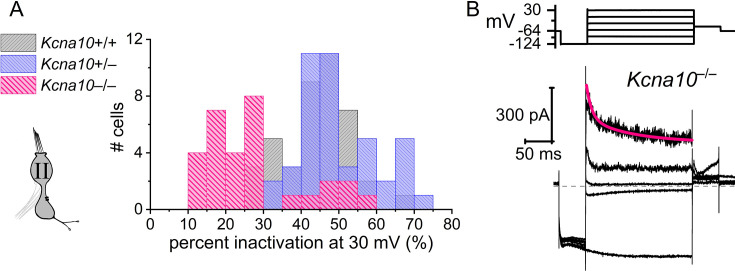
A minority of extrastriolar *Kcna10*^–/–^ type II hair cells (HCs) had a very small fast-inactivating outward rectifier current. (**A**) All extrastriolar Kcna10^+/+,+/–^ type II HCs inactivated by >30%. Most mature (>P12) extrastriolar Kcna10^–/–^ type II HCs inactivated by <30% but some inactivated by >30% (7/30, 23%) because they had fast inactivation (**B**). (**B**) Exemplar residual fast inactivation ($\tau_{Inact,Fast}$ = 10 ms at +30 mV). For the seven cells in this group, $\tau_{Inact,Fast}$ = 30 ± 6 ms, amplitude of fast inactivation = 310 ± 70 pA; activation peak *V*_half_ = –15 ± 2 mV and slope factor = 12.4 ± 0.9 mV. These parameters are similar to g_A_ but for the much smaller conductance (one-way ANOVAs).

**Table 3.** Type II hair cell K_V_ currents: activation and inactivation time course at +30 mV. Mean ± SEM. g is effect size, Hedge’s g. KWA is Kruskal–Wallis ANOVA.

Zone	*Kcna10*	$\tau_{Act}$ at 30 mV, ms^*, †^	$\tau_{Inact,Fast}$ at 30 mV, ms ^‡^^,^ ^§^	Fast inactivation prominence^¶^	Inactivation %^**^^,††^	*N* cells	Age (median, range)
LES	+/+	2.11 ± 0.09	23 ± 3	0.46 ± 0.03	45 ± 2	30	46, 14–312
	+/–	1.64 ± 0.09	15 ± 2	0.53 ± 0.03	51 ± 2	27	29, 13–280
	–/–	4.4 ± 0.5	NA	NA	25 ± 3	21	128, 15–355
MES	+/+	2.8 ± 0.5	50 ± 10	0.40 ± 0.04	42 ± 3	9	94, 22–296
	+/–	2.2 ± 0.2	90 ± 60	0.42 ± 0.03	47 ± 2	15	24, 13–52
	–/–	10 ± 7	NA	NA	29 ± 5	10	84, 28–355
Striola	+/+	2.7 ± 0.3	50 ± 10	0.31 ± 0.07	39 ± 3	5	45, 40–287
	+/–	2.9 ± 0.4	300 ± 200	0.3 ± 0.06	28 ± 2	5	19, 14–30
	–/–	7 ± 2	NA	NA	22 ± 2	6	202, 29–298

* –/– vs +/+: KWA, p = 0.0048, g 0.6; –/– vs +/–: KWA, p = 2.3E−7, g 0.6.

† Striola vs LES: KWA, p = 5.7E−4, g 1.0.

‡ +/– vs +/+: KWA, p = 0.027, g 0.2.

§ LES vs MES: KWA, p = 0.0018, g 0.3; LES vs Striola: KWA, p = 1.9E−4, g 0.8.

¶ LES vs Striola: two-way ANOVA, p = 0.0041, g 0.7.

** –/– vs +/+: two-way ANOVA, p < 1E−9, g 1.7; –/– vs +/–: two-way ANOVA, p < 1E−9, g 1.8.

†† Striola vs LES: two-way ANOVA, p = 3.4E−5, g 0.9; Striola vs MES: two-way ANOVA, p = 0.0011, g 1.0; interaction between genotype and zone: two-way ANOVA, p = 0.026.

To show voltage dependence of activation, we generated *G*–*V* curves for peak currents (sum of A-current and delayed rectifier) and steady-state currents measured at 200 ms, after g_A_ has mostly inactivated (Figure 3D.2). *Kcna10*^+/–^ HCs had smaller currents than *Kcna10*^+/+^ HCs, reflecting a smaller g_DR_ (Figure 3D) and faster fast inactivation (Figure 3A.3). As discussed later, these effects may relate to effects of the *Kcna10* gene dosage on the relative numbers of different K_V_1.8 heteromers.

For *Kcna10*^+/+^ and *Kcna10*^+/–^ HCs, the voltage dependence as summarized by *V*_half_ and slope factor (*S*) was similar. Relative to g_SS_, g_Peak_ had a more positive *V*_half_ (~–21 vs ~–26) and greater *S* (~12 vs ~9, Figure 3D, Table 4). Because g_Peak_ includes channels with and without fast inactivation, the shallower g_Peak_–*V* curve may reflect a more heterogeneous channel population. Only g_Peak_ showed zonal variation, with more positive *V*_half_ in LES than striola (~–20 vs ~–24 mV, Figure 3D, Table 4). We later suggest that variable subunit composition may drive zonal variation in g_Peak_.

**Table 4.** Type II hair cell K_V_ currents: activation voltage dependence. Mean ± SEM. g is effect size, Hedge’s g. KWA is Kruskal–Wallis ANOVA.

Zone	*Kcna10*	Peak *V*_1/2_, mV**	Peak *S*, mV^†^^†^, ^‡^	A-type g_max_/*C*_m_, nS/pF^§^ ^§^	SS *V*_half_, mV^¶^ ^¶^	SS *S*, mV****	SS g_max_/*C*_m_, nS/pF ^††^ ^‡ ‡^	*N* cells	Age (median, range)
LES	+/+	–19.8 ± 0.6	11.8 ± 0.4	4.0 ± 0.3	–25.0 ± 0.5	8.7 ± 0.3	7.1 ± 0.8	37	46, 14–312
	+/–	–19.8 ± 0.8	12.8 ± 0.4	3.8 ± 0.3	–26.8 ± 0.8	8.7 ± 0.3	4.9 ±0.4	35	29, 13–280
	–/–	–18 ± 1	11.7 ± 0.4	0.37 ± 0.05	–19 ± 1	12.1 ± 0.5	1.8 ±0.2	20	128, 15–355
MES	+/+	–22 ± 1	11 ± 0.7	4.1 ± 0.7	–26 ± 1	8.3 ± 0.5	9 ±1	11	94, 22–296
	+/–	–21 ± 1	11.8 ± 0.4	3.6 ± 0.5	–27 ± 1	9.0 ± 0.3	5.9 ±0.7	16	24, 13–52
	–/–	–19 ± 1	10.8 ± 0.6	0.6 ± 0.1	–20 ± 1	10.7 ± 0.7	2.5 ±0.3	15	84, 28–355
Striola	+/+	–24 ± 1	9.6 ± 0.5	5 ± 1	–26.6 ± 0.9	8.2 ± 0.4	12 ±1	7	45, 40–287
	+/–	–25 ± 2	9.4 ± 0.4	2.6 ± 0.6	–28 ± 2	8.2 ± 0.3	10±2	6	19, 14–30
	–/–	–21.3 ± 0.9	10.3 ± 0.5	0.7 ± 0.1	–21.7 ± 0.8	10.5 ± 0.6	3.9±0.5	8	202, 29–298

* Striola vs LES: two-way ANOVA, p = 0.00116, g 0.9.

† Striola vs MES: two-way ANOVA, p = 0.016, g 0.8; Striola vs LES: two-way ANOVA, p = 7.5E−6, g 1.2.

‡ –/– vs +/–: two-way ANOVA, p = 0.036, g 0.5.

§ –/– vs +/+: Welch ANOVA, p < 1E−9, g 2.3; –/– vs +/–: Welch ANOVA, p < 1E−9, g 2.3.

¶ –/– vs +/+: two-way ANOVA, p < 1E−9, g 1.4; –/– vs +/–: two-way ANOVA, p < 1E−9, g 1.6.

** –/– vs +/+: two-way ANOVA, p < 1E−9, g 1.4; –/– vs +/–: two-way ANOVA, p = 4.5E−7, g 1.1.

†† –/– vs +/+: Welch ANOVA, p < 1E−9, g 1.6; –/– vs +/–: Welch ANOVA, p < 1E−9, g 1.3; +/+vs +/–: Welch ANOVA, p = 0.007, g 1.6.

‡ ‡ Striola vs LES: one-way ANOVA, p = 0.001, g (0.9); Striola vs MES: one-way ANOVA, p = 0.01, g 0.8.

***Kcna10***^***–/–***^ ***type II HCs***. Kcna10^–/–^ type II HCs from all zones were missing g_A_ and 30–50% of g_DR_ (Figure 3B–D). The residual delayed rectifier (1.3 nS/pF) had a more positive *V*_half_ than g_DR_ in *Kcna10*^+/+,+/–^ HCs (~–20 vs ~–26 mV, Figure 3D.2). We refer to the K_V_1.8-dependent delayed rectifier component as g_DR_(K_V_1.8) and to the residual, K_V_1.8-independent delayed rectifier component as g_DR_(K_v_7) because, as we show later, it includes K_V_7 channels.

Figure 3—figure supplement 2 shows the development of K_V_1.8-dependent and -independent K_V_ currents in type II HCs with age from P5 to over P300. In *Kcna10*^+/+,+/–^ type II HCs, g_A_ was present at all ages with a higher % inactivation after P18 than at P5–P10 (Figure 3—figure supplement 2A.4). g_Peak_ did not change much above P12 except for a compression of conductance density from P13 to P370 (partial correlation coefficient = –0.4, p = 2E−5, Figure 3—figure supplement 2A.3).

We saw small rapidly inactivating outward currents in a minority of *Kcna10*^–/–^ type II HCs (23%, 7/30), all >P12 and extrastriolar (Figure 3—figure supplement 3). These currents overlapped with g_A_ in percent inactivation, inactivation kinetics, and activation voltage dependence but were very small. As discussed later, we suspect that these currents flow through homomers of inactivating K_V_ subunits that in control HCs join with K_V_1.8 subunits and confer inactivation on the heteromeric conductance.

### K_V_1.8 affects type II passive properties and responses to current steps

**Figure 4.**
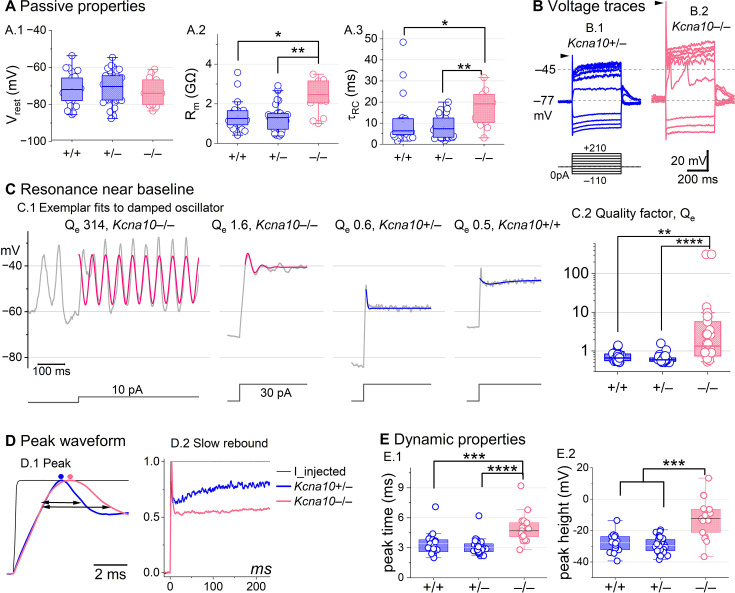
*Kcna10*^–/–^ type II hair cells (HCs) had larger, slower voltage responses and more electrical resonance. (**A**) Passive membrane properties near resting membrane potential: (**A.1**) Resting potential. *R*_input_ (**A.2**) and $\tau_{RC}$ (**A.3**) were obtained from single-exponential fits to voltage responses <15 mV. *R*_input_ and $\tau_{RC}$ were higher in *Kcna10*^–/–^ (n=13) than *Kcna10*^+/+^ (n=22, KWA p=0.015; p=0.016) and *Kcna10*^+/–^ (n=33, KWA p=0.002; p=0.008; see Table 5). (**B**) Exemplar voltage responses to iterated current steps (*bottom*) illustrate key changes in gain and resonance with K_V_1.8 knockout. (**B.1**) *Kcna10*^+/–^ type II HC (P24, LES) and (**B.2**) *Kcna10*^–/–^ type II HC (P53, LES). *Arrowheads,* depolarizing transients. (**C**) Range of resonance illustrated for *Kcna10*^–/–^ type II HCs (*left, pink curves fit to* Equation 5) and controls (*right, blue fits*). (**C.1**) *Resonant frequencies, left to right:* 19.6, 18.4, 34.4, and 0.3 Hz. Leftmost cell resonated spontaneously (before step). (**C.2**) Tuning quality (*Q*_e_; Equation 6) was higher for *Kcna10*^–/–^ (n=26) type II HCs (KWA: p = 0.0064 vs *Kcna10*^+/+^, n=23; p = 7E-8 vs *Kcna10*^+/–^, n=45). (**D**) *Kcna10*^–/–^ type II HCs had higher, slower peaks and much slower rebound potentials in response to large (170 pA) current steps. (**D.1**) Normalized to show initial depolarizing transient (*filled circles*, times of peaks; *horizontal arrows*, peak width at half-maximum). (**D.2**) Longer time scale to highlight how null mutation reduced post-transient rebound. (**E**) In *Kcna10*^–/–^ HCs (n=19), depolarizing transients evoked by a +90 pA step were slower to peak (**E.1**) than in *Kcna10*^+/+^ (n=19, 2-way ANOVA Tukey’s p<1E-9) and *Kcna10*^+/–^ (n=34, 2-way ANOVA Tukey’s p<1E-9) and (**E.2**) larger than in *Kcna10*^+/+^ (n=19, KWA p=0.006) and *Kcna10*^+/–^ (n=34, KWA p=2E-4). *Asterisks*: *p < 0.05; **p < 0.01; ***p < 0.001; and ****p < 0.0001. *Line,* median; *Box,* interquartile range; *Whiskers*, outliers.

In type II HCs, absence of K_V_1.8 did not change *V*_rest_ (Figure 4A.1) because g_A_ and g_DR_ both activate positive to rest, but significantly increased *R*_in_ and $\tau_{RC}$ (Figure 4A.2 and A.3).

**Table 5.** Type II hair cell passive membrane properties in the extrastriola (ES) and striola (S). Mean ± SEM (number of cells). g is effect size, Hedge’s g. KWA is Kruskal–Wallis ANOVA. Peak height and time were measured from responses to 170 pA input from rest.

Zone	Kcna10	*V*_rest_, mV	*R*_input_, GΩ*	$\tau_{RC}$, ms^†^	Peak height, mV^‡^	Peak time, ms^§^	*C*_m_, pF	Age (median, range)
ES	+/+	–71 ± 2 (19)	1.4 ± 0.2 (16)	11 ± 3 (16)	–20 ± 2 (15)	2.5 ± 0.2 (15)	4.7 ± 0.2 (50)	45, 16–312
	+/–	–71 ± 2 (34)	1.2 ± 0.1 (27)	9 ± 1 (27)	–20 ± 1 (30)	2.44 ± 0.08 (30)	4.6 ± 0.1 (52)	27, 13–280
	–/–	–76 ± 2 (9)	2.3 ± 0.3 (7)	16 ± 3 (7)	2 ± 6 (7)	3.6 ± 0.3 (7)	4.6 ± 0.2 (35)	53, 15–154
S	+/+	–73.1 ± 1.0 (6)	1.4 ± 0.1 (6)	9 ± 1 (6)	–20 ± 2 (5)	2.7 ± 0.1 (5)	4.6 ± 0.2 (7)	45, 40–224
	+/–	–71 ± 3 (5)	1.4 ± 0.3 (6)	7 ± 2 (6)	–20 ± 2 (6)	2.3 ± 0.1 (6)	4.8 ± 0.2 (6)	19, 19–30
	–/–	–68 ± 2 (6)	3.0 ± 0.7 (6)	26 ± 10 (6)	2 ± 7 (4)	4 ± 1 (4)	4.4 ± 0.3 (7)	178, 29–298

* –/– vs +/+: KWA, p = 0.015, g 1.2; –/– vs +/–: KWA, p = 0.002, g 1.5.

† –/– vs +/+: KWA, p = 0.016, g 0.7; –/– vs +/–: KWA, p = 0.008, g 1.2.

‡ –/– vs +/+: KWA, p = 0.006, g 2.1; –/– vs +/–: KWA, p = 2E−4, g 2.6.

§ –/– vs +/+: two-way ANOVA, p < 1E−9, g 1.3; –/– vs +/–: two-way ANOVA, p < 1E−9, g 1.9.

Positive current steps evoked an initial depolarizing transient in both *Kcna10*^+/+^ and *Kcna10*^–/–^ type II HCs, but the detailed time course differed (Figure 4B). Both transient and steady-state responses were larger in *Kcna10*^–/–^, consistent with their larger *R*_in_ values.

Absence of K_V_1.8 increased the incidence of sharp electrical resonance in type II HCs. Electrical resonance, which manifests as ringing responses to current steps, can support receptor potential tuning (Ashmore, 1983; Fettiplace, 1987; Hudspeth and Lewis, 1988; Ramanathan and Fuchs, 2002). Larger *R*_in_ values made *Kcna10*^–/–^ type II HCs more prone to electrical resonance; Figure 4C.1 shows a striking example. Median resonance quality (*Q*_e_, sharpness of tuning) was greater in *Kcna10*^–/–^ (1.33, n=26) than *Kcna10*^+/+^ (0.66, *n* = 23) or *Kcna10*^+/–^ (0.59, *n* = 44) type II HCs.

K_V_1.8 affected the time course of the initial peak in response to much larger current injections (Figure 4D, E). Fast activation of g_A_ in control type II HCs rapidly repolarizes the membrane and then inactivates, allowing the constant input current to progressively depolarize the cell, producing a slow rebound (Figure 4D.2). This behavior has the potential to counter mechanotranduction adaptation (Vollrath and Eatock, 2003).

### K_V_1.8 immunolocalized to basolateral membranes of both type I and II HCs

**Figure 5.**
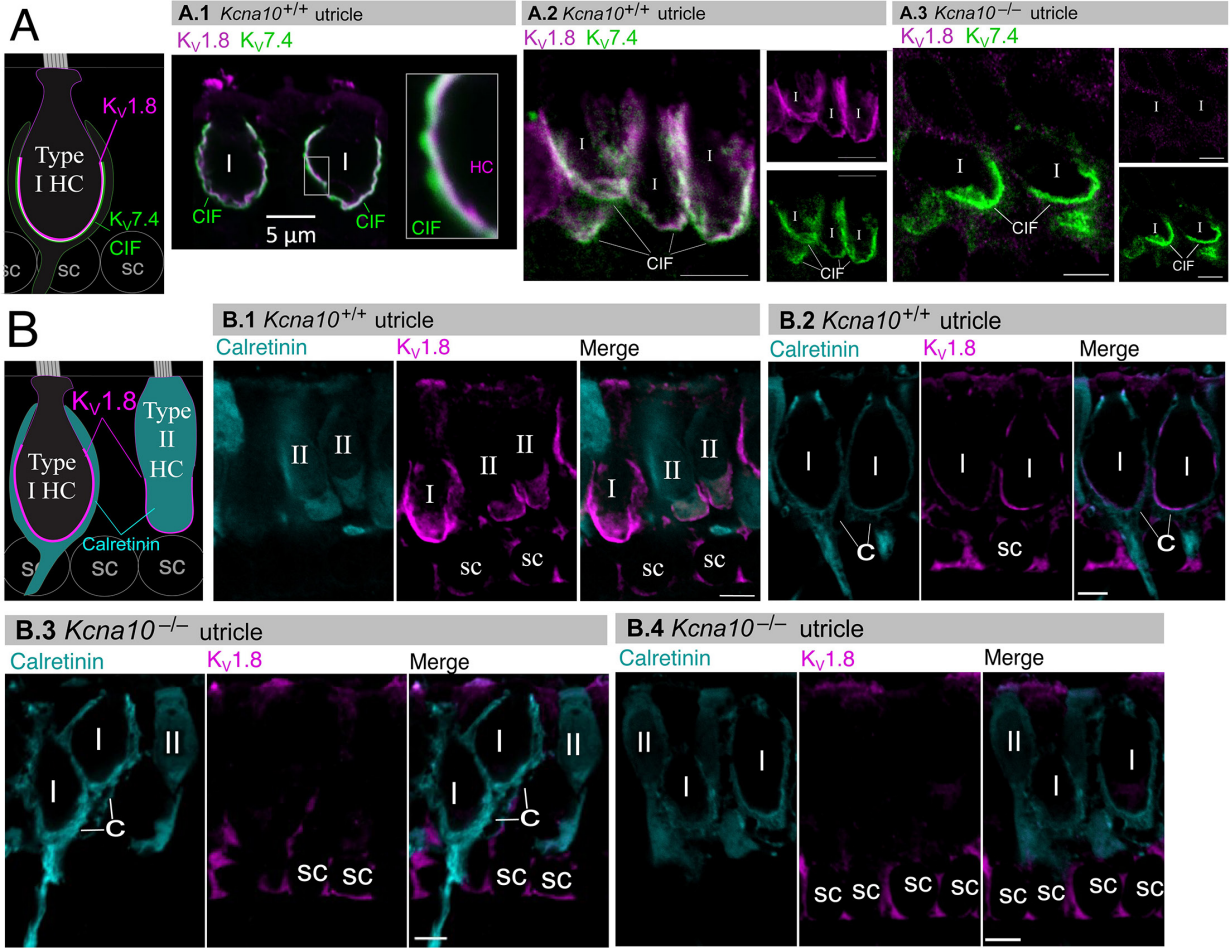
Type I and II hair cell (HC) basolateral membranes show specific immunoreactivity to Kv1.8 antibody (magenta). Antibodies for K_V_7.4 (A, green) and calretinin (B, cyan) were used as counterstains for calyx membrane (Kv7.4), type II HC cytoplasm (calretinin) and cytoplasm of striolar calyx-only afferents (calretinin). (**A**) *Left*, Cartoon showing K_V_7.4 on the calyx inner face membrane (CIF) and K_V_1.8 on the type I HC membrane. SC, supporting cell nuclei. *A.1–3*, Adult mouse utricle sections. K_V_7.4 antibody labeled calyces on two K_V_1.8-positive type I HCs (***A.1***), four K_V_1.8-positive type I HCs (***A.2***), and two K_V_1.8-negative type I HCs from a *Kcna10*^–/–^ mouse (***A.3***). (**B**) *Left*, Cartoon showing cytoplasmic calretinin stain in calyx-only striolar afferents and most type II HCs, and K_V_1.8 on membranes of both HC types. In wildtype utricles, K_V_1.8 immunolocalized to basolateral membranes of type I and II HCs (extrastriola, ***B.1***). K_V_1.8 immunolocalized to type I HCs (striola, ***B.2***). Staining of supporting cell (SC) membranes by Kv1.8 antibody was non-specific, as it was present in *Kcna10*^–/–^ tissue (striola, ***B.3*** and ***B.4***). All scale bars 5 µm.

If K_V_1.8 is a pore-forming subunit in the K_V_1.8-dependent conductances g_K,L_, g_A_, and g_DR_, it should localize to HC membranes. Figure 5 compares K_V_1.8 immunoreactivity in *Kcna10*^+/+^ and *Kcna10*^–/–^ utricles, showing specific immunoreactivity along the basolateral membranes of both HC types in *Kcna10*^+/+^ utricles. To identify HC type and localize the HC membrane, we used antibodies against K_V_7.4 (KCNQ4), an ion channel densely expressed in the calyceal ‘inner-face’ membrane next to the synaptic cleft (Hurley et al., 2006; Lysakowski et al., 2011), producing a cup-like stain around type I HCs (Figure 5A). K_V_1.8 immunoreactivity was present in HC membrane apposing K_V_7.4-stained calyx inner face in *Kcna10*^+/+^ utricles (Figure 5A.1 and A.2) and not in *Kcna10*^–/–^ utricles (Figure 5A.3).

In other experiments, we used antibodies against calretinin (CALB2), a cytosolic calcium-binding protein expressed by many type II HCs and also by striolar calyx-only afferents (Desai et al., 2005; Lysakowski et al., 2011, Figure 5B). An HC is type II if it is calretinin-positive (Figure 5B.1) or if it lacks a K_V_7.4- or calretinin-positive calyceal cup (Figure 5A.2 and B.3, rightmost cells). HC identification was confirmed with established morphological indicators: for example, type II HCs tend to have basolateral processes (feet) (Pujol et al., 2014) and, in the extrastriola, more apical nuclei than type I HCs.

Previously, Carlisle et al., 2012 reported K_V_1.8-like immunoreactivity in many cell types in the inner ear. In contrast, Lee et al., 2013 found that gene expression reporters indicated expression only in HCs and some supporting cells. Here, comparison of control and null tissue showed selective expression of HC membranes, and that some supporting cell staining is non-specific.

### K_V_1.4 may also contribute to g_A_

Results with the K_V_1.8 knockout suggest that type II HCs have an inactivating K_V_1 conductance that includes K_V_1.8 subunits. K_V_1.8, like most K_V_1 subunits, does not show fast inactivation as a heterologously expressed homomer (Lang et al., 2000; Ranjan et al., 2019; Dierich et al., 2020), nor do the K_V_1.8-dependent channels in type I HCs, as we show, and in cochlear inner HCs (Dierich et al., 2020). K_V_1 subunits without intrinsic inactivation can produce rapidly inactivating currents by associating with K_V_β1 or K_V_β3 subunits. K_V_β1 (*Kcnb1*) is present in type II HCs alongside K_V_β2 (*Kcnb2*) (McInturff et al., 2018; Jan et al., 2021; Orvis et al., 2021), which does not confer rapid inactivation (Dwenger et al., 2022).

Another possibility is that in type II HCs, K_V_1.8 subunits heteromultimerize with K_V_1.4 subunits—the only K_V_1 subunits which, when expressed as a homomer, have complete N-type (fast) inactivation (Stühmer et al., 1989). Multiple observations support this possibility. K_V_1.4 has been linked to g_A_ in pigeon type II HCs (Correia et al., 2008) and is the second-most abundant K_V_1 transcript in mammalian vestibular HCs, after K_V_1.8 (Scheffer et al., 2015). K_V_1.4 is expressed in type II HCs but not type I HCs (McInturff et al., 2018; Orvis et al., 2021), and is not found in striolar HCs (Jan et al., 2021; Orvis et al., 2021), where even in type II HCs, inactivation is slower and less extensive (Figure 3A).

**Figure 6.**
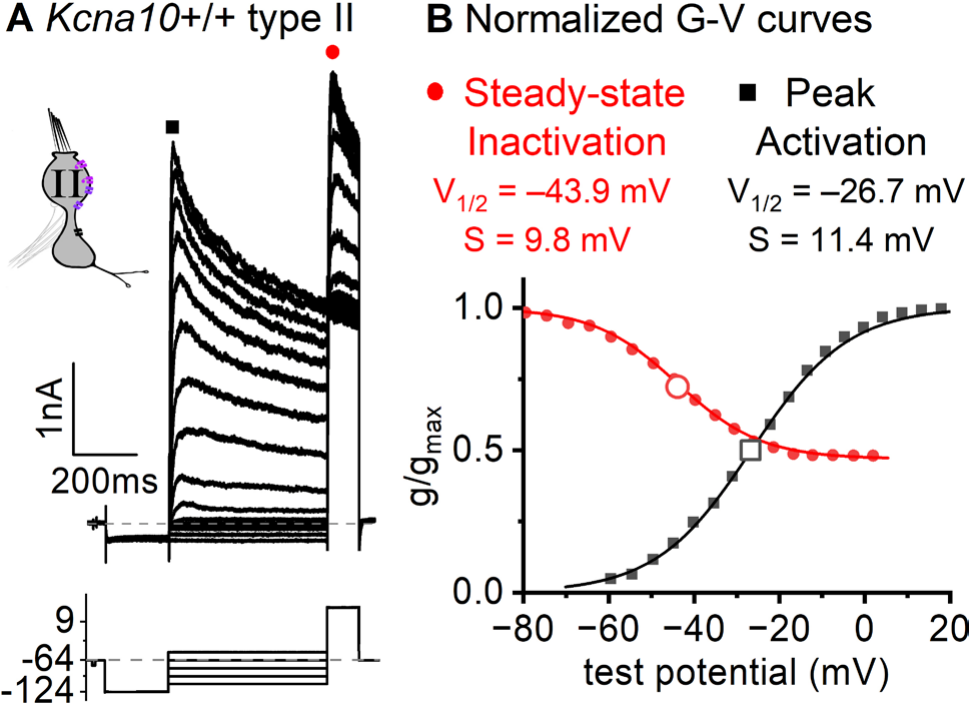
Inactivation curve of g_A_ in extrastriolar type II hair cells (HCs). (**A**) Modified voltage protocol measured accumulated steady-state inactivation at the tail potential. 100 μM ZD7288 in bath prevented contamination by HCN current. (**B**) Voltage dependence of g_A_’s steady-state inactivation (*h*_∞_ curve) and peak activation are consistent with K_V_1.4 heteromers. *Curves*, Boltzmann fits (Equation 1). *Average fit parameters* from *Kcna10*^+/+,+/–^ type II HCs, P40–P210, median P94. Inactivation: *V*_half_, –42 ± 2 mV (*n* = 11); *S*, 11 ± 1 mV. Activation: *V*_half_, –23 ± 1 mV (*n* = 11); *S*, 11.2 ± 0.4 mV.

Functional heteromers form between K_V_1.4 and other K_V_1.x and/or K_V_β1 (Imbrici et al., 2006; Correia et al., 2008; Al Sabi et al., 2011). Although K_V_1.4 and K_V_1.8 heteromers have not been studied directly, g_A_’s inactivation time course ($\tau_{Fast,Inact}$ of ~30 ms +30 mV, Figure 3A) and voltage dependence (*V*_half_ –41 mV, Figure 6B) are consistent with these other K_V_1.4-containing heteromers.

### K_V_7 channels contribute a small delayed rectifier in type I and II HCs

In *Kcna10*^–/–^ HCs, absence of *I*_K,L_ and *I*_A_ revealed smaller delayed rectifier K^+^ currents that, unlike *I*_K,L_, activated positive to resting potential and, unlike *I*_A_, lacked fast inactivation. Candidate channels include members of the K_V_7 (KCNQ, M-current) family, which have been identified previously in rodent vestibular HCs (Kharkovets et al., 2000; Rennie et al., 2001; Hurley et al., 2006; Scheffer et al., 2015).

**Figure 7.**
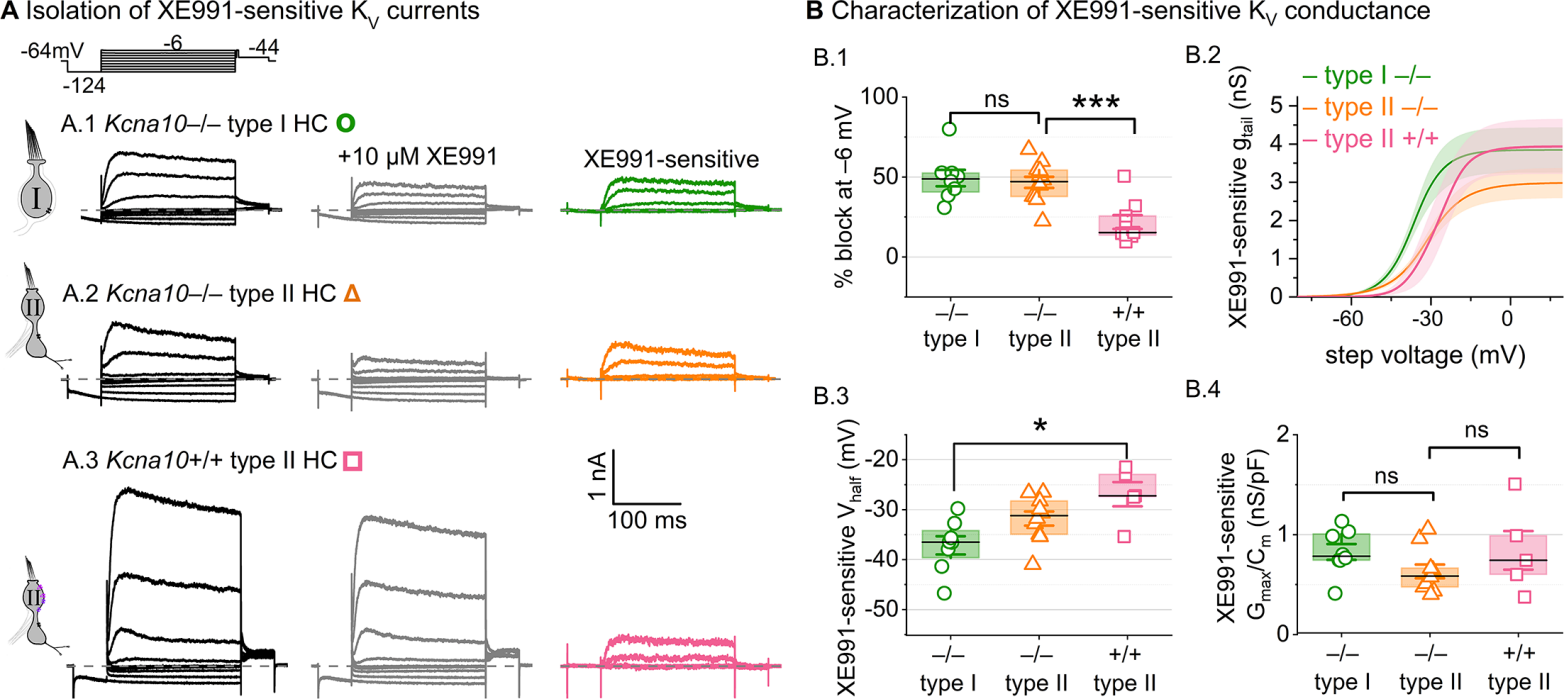
A K_V_7-selective blocker, XE991, reduced residual delayed rectifier currents in *Kcna10*^–/–^ type I and II hair cells (HCs). (**A**) XE991 (10 μM) partly blocked similar delayed rectifier currents in type I and II *Kcna10*^–/–^ HCs and a type II *Kcna10*^+/+^ HC. (**B**) Properties of XE991-sensitive conductance, _DR_(K_V_7). (**B.1**) % Block of steady-state current. (**B.2**) Mean tail *G*–*V* curves for *Kcna10*^–/–^ type I HCs (*n* = 8), *Kcna10*^–/–^ type II HCs (9), and *Kcna10*^+/+^ type II HCs (5); shading is ± SEM. (**B.3**) *V*_half_ was less negative in *Kcna10*^+/+^ type II than *Kcna10*^–/–^ type I HC (p = 0.01, KWA). (**B.4**) Conductance density was similar in all groups (ANOVA), non-significant at 0.4 power (*left*), 0.2 power (*right*). *Asterisks*: *p < 0.05 and ***p < 0.001. *Line,* median; *Box,* interquartile range; *Whiskers*, outliers.

We tested for K_V_7 contributions in *Kcna10*^–/–^ type I HCs, *Kcna10*^–/–^ type II HCs, and *Kcna10*^+/+,+/–^ type II HCs of multiple ages by applying XE991 at 10 µM (Figure 7A), a dose selective for K_V_7 channels (Brown et al., 2002) and close to the IC_50_ (Alexander et al., 2019). In *Kcna10*^–/–^ HCs of both types, 10 µM XE991 blocked about half of the residual K_V_ conductance (Figure 7B.1), consistent with K_V_7 channels conducting most or all of the non-K_V_1.8 delayed rectifier current. In all tested HCs (P8–355, median P224), the XE991-sensitive conductance did not inactivate substantially within 200 ms at any voltage, consistent with K_V_7.2, 7.3, 7.4, and 7.5 currents (Wang, 1998; Kubisch et al., 1999; Schroeder et al., 2000; Jensen et al., 2007; Xu et al., 2007). We refer to this component as g_DR_(K_V_7). The voltage dependence and g_max_ density (g_max_/*C*_m_) of g_DR_(K_V_7) were comparable across HC types and genotypes (Figure 7B.2–4). Although K_V_7.4 was not detectable in HCs during immunostaining (Figure 5), K_V_7.4 has been shown in type I HCs with immunogold labeling (Kharkovets et al., 2000; Hurley et al., 2006).

**Figure 7—figure supplement 1.**
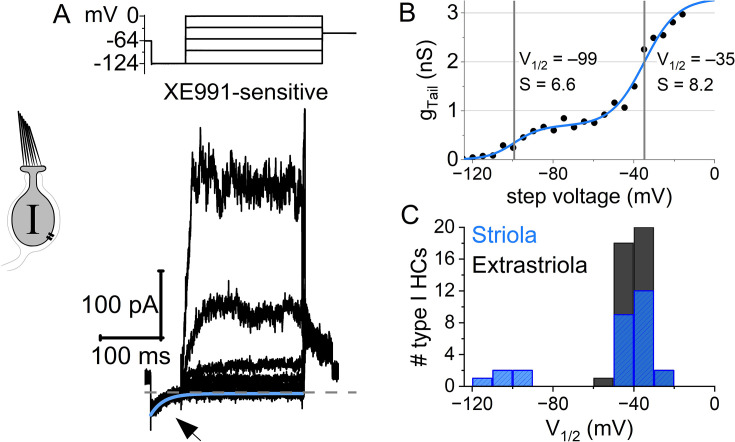
A minority of striolar *Kcna10*^–/–^ type I hair cells (HCs) had a small low-voltage-activated outward rectifier current in addition to a more positively activating outward rectifier. (**A**) Low-voltage-activated current from one cell was isolated by subtraction with 10 μM XE991 (P39), indicating that it was carried by K_V_7 channels. Deactivation of XE991-sensitive current evoked by step from –64 to –124 mV (*arrow*) was fit with exponential decay (τ = 21 ms). (**B**) XE991-sensitive tail *G*–*V* curve of the XE991-blocked conductance (*A*) was fit with a sum of two Boltzmann equations: G(*V*) = *A*_1_/(1 + exp((*V*_half,1_ – *V*)/*S*_1_)) + *A*_2_/(1 + exp((*V*_half,2_ – *V*)/*S*_2_)). (**C**) The low-voltage-activated *V*_half,1_ component was only seen in striolar *Kcna10*^–/–^ type I HCs, and even there in the minority: 5/23; 22%; P6–P370. It was always seen together with a more positively activating outward rectifier. Average Boltzmann parameters (n=5, including *B*): *A*_1_/(*A*_1_ + *A*_2_) = 0.15 ± 0.04, *V*_half,1_ = –106 ± 5 mV, *S*_1_ = 3.8 ± 0.8 mV, *V*_half,2_ = –41±1 mV, *S*_2_ = 7 ± 1 mV. Ages: P11, 39, 202, 202, 202. No extrastriolar type I HCs (0/45; P6–277) had a double activation tail *G*–*V* curve.

**Figure 7—figure supplement 2.**
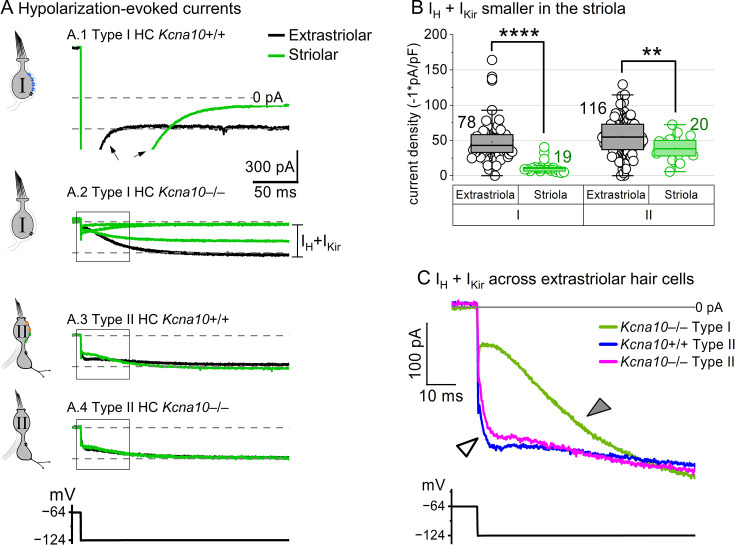
No difference was detected in H (HCN) and Kir (fast inward rectifier) currents between *Kcna10*^+/+^ and *Kcna10*^–/–^ hair cells (HCs), consistent with a specific involvement of K_V_1.8 in *Kcna10* expression. (**A**) Hyperpolarizing voltage steps evoked *I*_Kir_ and *I*_HCN_ in *Kcna10*^+/+,+/–,–/–^ type I and II HCs. (***A.1***) *Arrows*, deactivation of g_K,L_. (***A.2–A.4***) *Bracket,* the sum of *I*_H_ and *I*_Kir_ were measured as total inward current after 250 ms at –124 mV. (**B**) Summed *I*_KIR_ and *I*_H_ density was smaller in striola than extrastriola (type I HC KWA, p=4E-9; type II HC 2-way ANOVA Tukey’s p=0.006, see Supplementary file 1c). Data labels are number of cells. (**C**) Inward currents seen at the onset of hyperpolarization, including *I*_Kir_, were larger in type II HCs than type I. Magnification of boxed in inset from the extrastriolar cells in A.2–A.4. *Open arrowhead*, activation of fast inward rectifier, *I*_Kir_; *filled arrowhead*, slower activation of *I*_HCN_. *Asterisks*: **p < 0.01 and ****p < 0.0001. *Line,* median; *Box,* interquartile range; *Whiskers*, outliers.

These results are consistent with similar K_V_7 channels contributing a relatively small delayed rectifier in both HC types. In addition, the similarity of XE991-sensitive currents of *Kcna10*^+/+^ and *Kcna10*^–/–^ type II HCs indicates that knocking out K_V_1.8 did not cause general effects on ion channel expression. We did not test XE991 on *Kcna10*^+/+,+/–^ type I HCs because g_K,L_ runs down in ruptured patch recordings (Rüsch and Eatock, 1996a; Chen and Eatock, 2000; Hurley et al., 2006), which could contaminate the XE991-sensitive conductance obtained by subtraction.

In one striolar *Kcna10*^–/–^ type I HC, XE991 also blocked a small conductance that activated negative to rest (Figure 7—figure supplement 1A, B). This conductance (*V*_half_ ~ = –100 mV, Figure 7—figure supplement 1C) was detected only in *Kcna10*^–/–^ type I HCs from the striola (5/23 vs 0/45 extrastriolar). The *V*_half_ and $\tau_{deactivation}$ were similar to values reported for K_V_7.4 channels in cochlear HCs (Wong et al., 2004; Dierich et al., 2020). This very negatively activating K_V_7 conductance coexisted with the larger more positively activating K_V_7 conductance (Figure 7—figure supplement 1C) and was too small (<0.5 nS/pF) to contribute significantly to g_K,L_ (~10–100 nS/pF, Figure 1D).

### Other channels

While our data are consistent with K_V_1.8- and K_V_7-containing channels carrying most of the outward-rectifying current in mouse utricular HCs, there is evidence in other preparations for additional channels, including K_V_11 (KCNH, Erg) channels in rat utricular type I HCs (Hurley et al., 2006) and BK (KCNMA1) channels in rat utricle and rat and turtle semicircular canal HCs (Schweizer et al., 2009; Contini et al., 2020).

BK is expressed in mouse utricular HCs (McInturff et al., 2018; Jan et al., 2021; Orvis et al., 2021). However, Ca^2+^-dependent currents have not been observed in mouse utricular HCs, and we found little to no effect of the BK-channel blocker iberiotoxin at a dose (100 nM) well beyond the IC_50_: percent blocked at –30 mV was 2 ± 6% (3 *Kcna10*^–/–^ type I HCs); 1 ± 5% (5 *Kcna10*^+/+,+/–^ type II HCs); 7% and 14% (2 *Kcna10*^–/–^ type II HCs). We also did not see N-shaped *I*–*V* curves typical of many Ca^2+^-dependent K^+^ currents. In our ruptured-patch recordings, Ca^2+^-dependent BK currents and erg channels may have been eliminated by wash-out of the HCs’ small Ca_V_ currents (Bao et al., 2003) or cytoplasmic second messengers (Hurley et al., 2006).

To check whether the constitutive K_V_1.8 knockout has strong non-specific effects on channel trafficking, we examined the summed HCN and fast inward rectifier currents (*I*_H_ and *I*_Kir_) at –124 mV, and found them similar across genotypes (Figure 7—figure supplement 2). The g_K,L_ knockout allowed identification of zonal differences in *I*_H_ and *I*_Kir_ in type I HCs, previously examined in type II HCs (Masetto and Correia, 1997; Levin and Holt, 2012). In type I HCs from both control and null utricles, *I*_H_ and *I*_Kir_ were less prevalent in striola than extrastriola, and, when present, the combined inward current was smaller (Figure 7—figure supplement 2).

## Discussion

We have shown that constitutive knockout of K_V_1.8 eliminated g_K,L_ in type I HCs, and g_A_ and much of g_DR_ in type II HCs. K_V_1.8 immunolocalized specifically to the basolateral membranes of type I and II HCs. We conclude that K_V_1.8 is a pore-forming subunit of g_K,L_, g_A_, and part of g_DR_ [g_DR_(K_V_1.8)]. We suggest that fast inactivation of g_A_ may arise from heteromultimerization of non-inactivating K_V_1.8 subunits and inactivating K_V_1.4 subunits. Finally, we showed that a substantial component of the residual delayed rectifier current in both type I and II HCs comprises K_V_7 channels.

K_V_1.8 is expressed in HCs from mammalian cochlea (Dierich et al., 2020), avian utricle (Scheibinger et al., 2022), and zebrafish (Erickson and Nicolson, 2015). Our work suggests that in anamniotes, which lack type I cells and g_K,L_, K_V_1.8 contributes to g_A_ and g_DR_, which are widespread in vertebrate HCs (reviewed in Meredith and Rennie, 2016). K_V_1.8 expression has not been detected in rodent brain but is reported in the pacemaker nucleus of weakly electric fish (Smith et al., 2018).

### K_V_1.8 subunits may form homomultimers to produce g_K,L_ in type I HCs

Recent single-cell expression studies on mouse utricles (McInturff et al., 2018; Jan et al., 2021; Orvis et al., 2021) have detected just one K_V_1 subunit, K_V_1.8, in mouse type I HCs. Given that K_V_1.8 can only form multimers with K_V_1 family members, and given that g_K,L_ channels are present at very high density (~150 per μm^2^ in rat type I, Chen and Eatock, 2000), it stands to reason that most or all of the channels are K_V_1.8 homomers. Other evidence is consistent with this proposal. g_K,L_ (Rüsch and Eatock, 1996a) and heterologously expressed K_V_1.8 homomers in oocytes (Lang et al., 2000) are non-inactivating and blocked by millimolar Ba^2+^ and 4-aminopyridine and >10 mM tetraethyl ammonium. Unlike channels with K_V_1.1, K_V_1.2, and K_V_1.6 subunits, g_K,L_ is not sensitive to 10 nM α-dendrotoxin (Rüsch and Eatock, 1996a). g_K,L_ and heterologously expressed K_V_1.8 channels have similar single-channel conductances (~20 pS for g_K,L_ at positive potentials, Chen and Eatock, 2000; 11 pS in oocytes, Lang et al., 2000). g_K,L_ is inhibited—or positively voltage-shifted—by cGMP (Behrend et al., 1997; Chen and Eatock, 2000), presumably via the C-terminal cyclic nucleotide-binding domain of K_V_1.8.

A major novel property of g_K,L_ is that it activates 30–60 mV negative to type II K_V_1.8 conductances and most other low-voltage-activated K_V_ channels (Ranjan et al., 2019). The very negative activation range is a striking difference between g_K,L_ and known homomeric K_V_1.8 channels. Heterologously expressed homomeric K_V_1.8 channels have an activation *V*_half_ of –10 to 0 mV (*X. laevis* oocytes, Lang et al., 2000; Chinese hamster ovary cells, Dierich et al., 2020). In cochlear inner HCs, currents attributed to K_V_1.8 (by subtraction of other candidates) have a near-zero activation *V*_half_ (–4 mV, Dierich et al., 2020).

Possible factors in the unusually negative voltage dependence of g_K,L_ include:

(1) *Elevation of extracellular K^+^* by the enveloping calyceal terminal, unique to type I HCs (Lim et al., 2011; Contini et al., 2012; Spaiardi et al., 2020; Govindaraju et al., 2023). High K^+^ increases conductance though g_K,L_ channels (Contini et al., 2020), perhaps through K^+^-mediated relief of C-type inactivation (López-Barneo et al., 1993; Baukrowitz and Yellen, 1995). We note, however, that g_K,L_ is open at rest even in neonatal mouse utricles cultured without innervation (Rüsch et al., 1998) and persists in dissociated type I HCs (Chen and Eatock, 2000; Hurley et al., 2006).

(2) *The high density of g*_*K,L*_ (~50 nS/pF in striolar *Kcna10*^+/+^ HCs) implies close packing of channels, possibly represented by particles (12–14 nm) seen in freeze-fracture electron microscopy of the type I HC membrane (Gulley and Bagger-Sjöbäck, 1979; Sousa et al., 2009). Such close channel packing might hyperpolarize in situ voltage dependence of g_K,L_, as proposed for K_V_7.4 channels in outer HCs (Perez-Flores et al., 2020). Type I HC-specific partners that may facilitate this close packing include ADAM11 (McInturff et al., 2018), which clusters presynaptic K_V_1.1 and K_V_1.2 to enable ephaptic coupling at a cerebellar synapse (Kole et al., 2015).

(3) *Modulation by accessory subunits*. Type I HCs express K_V_β1 (McInturff et al., 2018; Orvis et al., 2021), an accessory subunit that can confer fast inactivation and hyperpolarize activation *V*_half_ by ~10 mV. K_V_β1 might interact with K_V_1.8 to shift voltage dependence negatively. Arguments against this possibility include that g_K,L_ lacks fast inactivation (Rüsch and Eatock, 1996a; Hurley et al., 2006; Spaiardi et al., 2017) and that cochlear inner HCs co-express K_V_1.8 and K_V_β1 (Liu et al., 2018) but their K_V_1.8 conductance has a near-0 *V*_half_ (Dierich et al., 2020).

### K_V_1.8 subunits may combine with different subunits to produce g_A_ and K_V_1.8-dependent g_DR_ in type II HCs

The K_V_1.8-dependent conductances of type II HCs vary in their fast and slow inactivation. In not showing fast inactivation (Lang et al., 2000; Ranjan et al., 2019; Dierich et al., 2020), heterologously expressed K_V_1.8 subunits resemble most other K_V_1 family subunits, with the exception of K_V_1.4 (for comprehensive review, see Ranjan et al., 2019). K_V_1.4 is a good candidate to provide fast inactivation based on immunolocalization and voltage dependence (Figures 4 and 6). We suggest that g_A_ and g_DR_(K_V_1.8) are K_V_1.8-containing channels that may include a variable number of K_V_1.4 subunits and K_V_β2 and K_V_β1 accessory subunits.

K_V_1.4–K_V_1.8 heteromeric assembly could account for several related observations. The faster $\tau_{Inact,Fast}$ in *Kcna10*^+/–^ relative to *Kcna10*^+/+^ type II HCs (Figure 3A.3, Figure 3—figure supplement 1A.1) could reflect an increased ratio of K_V_1.4–K_V_1.8 subunits and therefore more N-terminal inactivation domains per heteromeric channel. Zonal variation in the extent and speed of N-type inactivation (Figure 3A) might arise from differential expression of K_V_1.4. The small fast-inactivating conductance in ~20% of extrastriolar *Kcna10*^–/–^ type II HCs (Figure 3—figure supplement 3) might flow through K_V_1.4 homomers.

Fast inactivation may also receive contribution from K_V_β subunits. K_V_β1 is expressed in type II HCs (McInturff et al., 2018; Jan et al., 2021; Orvis et al., 2021), and, together with K_V_1.4, has been linked to g_A_ in pigeon vestibular HCs (Correia et al., 2008). K_V_β2, also expressed in type II HCs (McInturff et al., 2018; Orvis et al., 2021), accelerates but does not confer fast inactivation.

We speculate that g_A_ and g_DR_(K_V_1.8) have different subunit composition: g_A_ may include heteromers of K_V_1.8 with other subunits that confer rapid inactivation, while g_DR_(K_V_1.8) may comprise homomeric K_V_1.8 channels, given that they do not have N-type inactivation.

### K_V_1.8 relevance for vestibular function

In both type I and II utricular HCs, K_V_1.8-dependent channels strongly shape receptor potentials in ways that promote temporal fidelity rather than electrical tuning (Lewis, 1988), consistent with the utricle’s role in driving reflexes that compensate for head motions as they occur. This effect is especially pronounced for type I HCs, where the current-step evoked voltage response reproduces the input with great speed and linearity (Figure 2).

g_K,L_dominates passive membrane properties in mature *Kcna10*^+/+,+/–^ type I HCs such that *Kcna10*^–/–^ type I HCs are expected to have receptor potentials with higher amplitudes but lower low-pass corner frequencies, closer to those of type II HCs and immature HCs of all types (Correia et al., 1996; Rüsch and Eatock, 1996a; Songer and Eatock, 2013). In *Kcna10*^–/–^ epithelia, we expect the lack of a large basolateral conductance open at rest to reduce the speed and gain of non-quantal transmission, which depends on K^+^ ion efflux from the type I HC to change electrical and K^+^ potentials in the synaptic cleft (Govindaraju et al., 2023). In HCs, K^+^ enters the mechanosensitive channels of the hair bundle from the K^+^-rich apical endolymph and exits through basolateral potassium conductances into the more conventional low-K^+^ perilymph. For the type I-calyx synapse, having in the HC a large, non-inactivating K^+^ conductance open across the physiological range of potentials avoids channel gating time and allows for instantaneous changes in current into the cleft and fast afferent signaling (Pastras et al., 2023).

In contrast, mature type II HCs face smaller synaptic contacts and have K_V_1.8-dependent currents that are not substantially activated at resting potential. They do affect the time course and gain of type II HC responses to input currents, speeding up depolarizing transients, producing a repolarizing rebound during the step, and reducing resonance.

Type I and II vestibular HCs are closely related, such that adult type II HCs acquire type I-like properties upon deletion of the transcription factor *Sox2* (Stone et al., 2021). In normal development of the two cell types, the *Kcna10* gene generates biophysically distinct and functionally different ion channels, presenting a natural experiment in functional differentiation of sensory receptor cells.

## Materials and methods

**Key resources table:** 

Reagent type (species) or resource	Designation	Source or reference	Identifiers	Additional information
Antibody	Anti-Kv1.8 (Rabbit polyclonal)	Alomone	Cat# APC-157, lot# 0102, RRID:AB_2341039	1:200 or 1:400
Antibody	Anti-calretinin (goat polyclonal)	Millipore	Cat# AB1550, lot# 9669, RRID:AB_90764	1:600
Antibody	Anti-Kv7.4 (mouse IgG1 monoclonal)	NeuroMab	Cat# 2HK-65, RRID:AB_2131828	1:200
Peptide, recombinant protein	Iberiotoxin	Alomone	STI-400	100 nM (water)
Chemical compound, drug	XE991	Sigma	X2254	100 µM (water)
Chemical compound, drug	ZD7288	Tocris	APN18035-2	100 µM (water)
Peptide, recombinant protein	Bovine serum albumin	Fisher	BP671	1 mg/ml (water)

### Preparation

All procedures for handling animals followed the NIH Guide for the Care and Use of Laboratory Animals and were approved by the Institutional Animal Care and Use Committees of the University of Chicago (Animal Care and Use Procedure #72360) and the Office of Animal Care and Institutional Biosafety at the University of Illinois Chicago (Protocol for Animal Use #17106). Most mice belonged to a transgenic line with a knockout allele of *Kcna10* (referred to here as *Kcna10*^–/–^). Our breeding colony was established with a generous gift of such animals from Sherry M. Jones and Thomas Friedman. These animals are described in their paper (Lee et al., 2013). Briefly, the Texas A&M Institute for Genomic Medicine generated the line on a C57BL/6;129SvEv mixed background by replacing Exon 3 of the *Kcna10* gene with an IRES-bGeo/Purocassette. Mice in our colony were raised on a 12:12 hr light–dark cycle with access to food and water ad libitum.

Semi-intact utricles were prepared from ~150 male and ~120 female mice, postnatal days (P) 5–375, for same-day recording. HC K_V_ channel data were pooled across sexes as most results did not appear to differ by sex; an exception was that g_K,L_ had a more negative *V*_half_ in males (Supplementary file 1a), an effect not clearly related to age, copy number, or other properties of the activation curve.

Preparation, stimulation, and recording methods followed our previously described methods for the mouse utricle (Vollrath and Eatock, 2003). Mice were anesthetized through isoflurane inhalation. After decapitation, each hemisphere was bathed in ice-cold, oxygenated Liebowitz-15 (L15) media. The temporal bone was removed, the labyrinth was cut to isolate the utricle, and the nerve was cut close to the utricle. The utricle was treated with proteinase XXIV (100 μg/ml, ~10 min, 22°C) to facilitate removal of the otoconia and attached gel layer and mounted beneath two glass rods affixed at one end to a coverslip.

### Electrophysiology

We used the HEKA Multiclamp EPC10 with Patchmaster acquisition software, filtered by the integrated HEKA filters: a 6-pole Bessel filter at 10 kHz and a second 4-pole Bessel filter at 5 kHz, and sampled at 10–100 kHz. Recording electrodes were pulled (PC-100, Narishige) from soda lime glass (King’s Precision Glass R-6) wrapped in paraffin to reduce pipette capacitance. Internal solution contained (in mM) 135 KCl, 0.5 MgCl_2_, 3 MgATP, 5 4-(2-hydroxyethyl)piperazine-1-ethane-sulfonic acid (HEPES), 5 ethylene glycol tetraacetic acid (EGTA), 0.1 CaCl_2_, 0.1 Na-cAMP, 0.1 LiGTP, 5 Na_2_CreatinePO_4_ adjusted to pH 7.25 and ~280 mmol/kg by adding ~30 mM KOH. External solution was Liebowitz-15 media supplemented with 10 mM HEPES (pH 7.40, 310 ± 10 mmol/kg). Recording temperature was 22–25°C. Pipette capacitance and membrane capacitance transients were subtracted during recordings with Patchmaster software. Series resistance (8–12 MΩ) was measured after rupture and compensated 60–80% with the amplifier, to final values of ~2 MΩ. Potentials are corrected for remaining (uncompensated) series resistance and liquid junction potential of ~+4 mV, calculated with LJPCalc software (Marino et al., 2014).

*Kcna10*^–/–^ HCs appeared healthy in that cells had resting potentials negative to –50 mV, cells lasted a long time (20–30 min) in ruptured patch recordings, membranes were not fragile, and extensive blebbing was not seen. Type I HCs with g_K,L_ were transiently hyperpolarized to ~–90 mV to close g_K,L_ enough to increase *R*_input_ above 100 MΩ, as needed to estimate series resistance and cell capacitance. The average resting potential, *V*_rest_, was –87 mV ±1 (41), similar to the calculated *E*_K_ of –86.1 mV, which is not surprising given the large K^+^ conductance of these cells. *V*_rest_ is likely more positive in vivo, where lower endolymphatic Ca^2+^ increases standing inward current through MET channels.

Voltage protocols to characterize K_V_ currents differed slightly for type I and II HCs. In standard protocols, the cell is held at a voltage near resting potential (–74 mV in type I and –64 mV in type II), then jumped to –124 mV for 200 ms in type I HCs in order to fully deactivate g_K,L_ and 50 ms in type II HCs in order to remove baseline inactivation of g_A_. The subsequent iterated step depolarizations lasted 500 ms in type I HCs because g_K,L_ activates slowly (Wong et al., 2004) and 200 ms in type II HCs, where K_V_ conductances activate faster. The 50 ms tail voltage was near the reversal potential of HCN channels (–44 mV in mouse utricular HCs, Rüsch et al., 1998) to avoid HCN current contamination.

*G*–*V* (activation) parameters for control type I cells may be expected to vary across experiments on semi-intact (as here), organotypically cultured and denervated (Rüsch et al., 1998), or dissociated-cell preparations, reflecting variation in retention of the calyx (Discussion) and voltage step durations (Wong et al., 2004) which elevate K^+^ concentration around the HC. Nevertheless, the values we obtained for type I and II HCs resemble values recorded elsewhere, including experiments in which extra care was taken to avoid extracellular K^+^ accumulation (Spaiardi et al., 2017; Spaiardi et al., 2020). The effects of K^+^ accumulation on g_K,L_’s steady-state activation curves are small because the operating range is centered on E and can be characterized with relatively small currents (Figure 1A).

### Pharmacology

Drug-containing solutions were locally with BASI Bee Hive syringes at a final flow rate of 20 μl/min and a dead time of ~30 s. Global bath perfusion was paused during drug perfusion and recording, and only one cell was used per utricle. Aliquots of test agents in solution were prepared, stored at –20°C, and thawed and added to external solution on the recording day (see Key Resources Table).

### Analysis

Data analysis was performed with software from OriginLab (Northampton, MA) and custom MATLAB scripts using MATLAB fitting algorithms.

### Fitting voltage dependence and time course of conductances

*G–V curves*. Current was converted to conductance (*G*) by dividing by driving force (*V* – *E*_K_; *E*_K_ calculated from solutions). For type I HCs, tail *G*–*V* curves were generated from current 1 ms after the end of the iterated voltage test step. For type II HCs, peak *G*–*V* curves were generated from peak current during the step and steady-state *G*–*V* curves were generated from current 1 ms before the end of a 200-ms step. We fit *G*–*V* curves to the first-order Boltzmann equation (Equation 1) using a custom MATLAB function (fitzmann.m, Source code 2).

(1) $$G\left(V\right)=G_{min}+\frac{G_{max}}{1+exp(\frac{V_{half}-V}{S})}$$

*V*_half_ is the midpoint, and *S* is the slope factor, inversely related to curve steepness near activation threshold.

*Activation time course of type II HCs*. We fit current traces using a custom MATLAB function (fitkin.m, Source code 1). For type II HCs lacking fast inactivation, outward current activation was fit with Equation 2.

(2) $$I\left(t\right)=I_{SS}*{\left(1-exp(-\frac{t}{\tau_{w}})\right)}^{n}+I_{0}$$

*I*_SS_ is steady-state current; $\tau_{w}$ is activation time constant (referred to elsewhere as $\tau_{Act}$); *n* is the state factor related to the number of closed states (typically constrained to 3); and *I*_o_ is baseline current.

To measure activation and inactivation time course of g_A_, we used Equation 3 to fit outward K^+^ currents evoked by steps from –125 mV to above –50 mV (Rothman and Manis, 2003).

(3) $$I\left(t\right)=I_{max}*{\left(1-exp(-\frac{t}{\tau_{w}})\right)}^{n}*\left[1-Z*\left(f*\left(1-exp(-\frac{t}{\tau_{zf}})\right)+\left(1-f\right)*\left(1-exp(-\frac{t}{\tau_{zs}})\right)\right)\right]+I_{0}$$

*Z* is total steady-state inactivation (0 ≤ *Z* < 1 means incomplete inactivation, which allows the equation to fit non-inactivating delayed rectifier currents); *f* is the fraction of fast inactivation relative to total inactivation; *I*_max_ is maximal current; $\tau_{zf}$ (referred to elsewhere as $\tau_{Inact,Fast}$) and $\tau_{zs}$ are the fast and slow inactivation time constants. We chose to compare fit parameters at 30 ± 2 mV (91), where fast and slow inactivation were consistently separable and g_A_ was maximized. In most *Kcna10*^–/–^ and some striolar *Kcna10*^+/+,+/–^ cells, where fast inactivation was absent and adjusted *R*^2^ did not improve on a single-exponential fit by >0.01, we constrained *f* in Equation 3 to 0 to avoid overfitting.

For *Peak G*–*V* relations, peak conductance was taken from fitted curves (Equations 2 and 3). To construct ‘*Steady-state*’ *G*–*V* relations, we used current at 200 ms, which was only 6 ± 1% (94) greater than steady-state estimated from fits to Equation 3 (Figure 3C, D).

Percent inactivation was calculated at 30 mV with Equation 4:

(4) $$\%\;Inactivation=\left(I_{Peak}-I_{SS}\right)/I_{Peak}$$

*I*_Peak_ is maximal current, and *I*_SS_ is current at the end of a 200-ms voltage step.

The electrical resonance of type II HCs was quantified by fitting voltage responses to current injection steps (Songer and Eatock, 2013). We fit Equation 5, a damped sinusoid, to the voltage trace from half-maximum of the initial depolarizing peak until the end of the current step.

(5) $$V(t)=V_{ss}+V_{p}*exp(-\frac{t}{\tau_{e}})*\sin\left(2\pi f_{e}-\theta \right)$$

*V*_SS_ is steady-state voltage; *V*_p_ is the voltage of the peak response; $\tau_{e}$ is the decay time constant; *f*_e_ is the fundamental frequency; and *θ* is the phase angle shift.

Quality factor, *Q*_e_, was calculated with Equation 6 (Crawford and Fettiplace, 1981).

(6) $$Q_{e}={\left[{\left(\pi f_{e}\tau_{e}\right)}^{2}+0.25\right]}^{1/2}$$

### Statistics

We give means ± SEM for normally distributed data, and otherwise, median and range. Data normality was assessed with the Shapiro–Wilk test for *n* < 50 and the Kolmogorov–Smirnov test for *n* > 50. To assess homogeneity of variance we used Levene’s test. With homogeneous variance, we used two-way ANOVA for genotype and zone with the post hoc Tukey’s test. When variance was non-homogeneous, we used one-way Welch ANOVA with the posthoc Games–Howell test. For data that were not normally distributed, we used the non-parametric one-way Kruskal–Wallis ANOVA (KWA) with posthoc Dunn’s test. Effect size is Hedge’s g (g). For age dependence, we used partial correlation coefficients controlling for genotype and zone. Statistical groups may have different median ages, but all have overlapping age ranges. In figures, asterisks represent p-value ranges as follows: *p < 0.05; **p < 0.01; ***p < 0.001; ****p < 0.0001.

### Immunohistochemistry

Mice were anesthetized with Nembutal (80 mg/kg), then perfused transcardially with 40 ml of physiological saline containing heparin (400 IU), followed by 2 ml/g body weight fixative (4% paraformaldehyde, 1% picric acid, and 5% sucrose in 0.1 M phosphate buffer at pH 7.4, sometimes with 1% acrolein). Vestibular epithelia were dissected in phosphate buffer, and tissues were cryoprotected in 30% sucrose-phosphate buffer overnight at 4°C. Otoconia were dissolved with Cal-Ex (Fisher Scientific) for 10 min. Frozen sections (35 μm) were cut with a sliding microtome. Immunohistochemistry was performed on free-floating sections. Tissues were first permeabilized with 4% Triton X-100 in phosphate-buffered saline (PBS) for 1 hr at room temperature, then incubated with 0.5% Triton X-100 in a blocking solution of 0.5% fish gelatin and 1% bovine serum albumin for 1 hr at room temperature. Sections were incubated with two to three primary antibodies for 72 hr at 4°C and with two to three secondary antibodies. Sections were rinsed with PBS between and after incubations and mounted on slides in Mowiol (Calbiochem).

## Data Availability

Data generated and analyzed in this study are available on Dryad (https://doi.org/10.5061/dryad.37pvmcvrw). Dryad hosts downloadable spreadsheets that are organized as follows: F1_sourcedata, numerical data for Figure 1; F1-fs1_sourcedata, numerical data for Figure 1-figure supplement 1; F2_sourcedata, numerical data for Figure 2; F3_sourcedata, numerical data for Figure 3; F3-fs1_sourcedata, numerical data for Figure 3—figure supplement 1; F3-fs2_sourcedata, numerical data for Figure 3—figure supplement 2; F3-fs3_sourcedata, numerical data for Figure 3—figure supplement 3; F4_sourcedata, numerical data for Figure 4; F6_sourcedata, numerical data for Figure 6; F7_sourcedata, numerical data for Figure 7; F7-fs1_sourcedata, numerical data for Figure 7—figure supplement 1; F7-fs2_sourcedata, numerical data for Figure 7—figure supplement 2. The following dataset was generated: MartinHR LysakowskiA EatockRA 2024The potassium channel subunit Kv1.8 (Kcna10) is essential for the distinctive outwardly rectifying conductances of type I and II vestibular hair cellsDryad Digital Repository10.5061/dryad.37pvmcvrwPMC1161438439625061

## Additional files

Supplementary file 1. This file contains descriptive and comparative statistics on three additional analyses of the dataset. (**a**) Test of sex differences in hair cell K_V_ channel data. (**b**) We did not detect a genotype effect on soma size of type I hair cells (HCs). (**c**) I_Kir_ and I_H­_ were greater in the extrastriola (ES) than striola (S), but did not vary by genotype.

MDAR checklist

Source code 1. Fitkin*.*m is a MATLAB function to fit the activation and inactivation kinetics of K_V_ currents to Equations 2; 3. Fitkin.m processed current–time traces and output parameters found in Figure 3, Figure 3—figure supplement 1, and Table 3.

Source code 2. Fitzmann*.*m is a MATLAB function to fit the activation voltage dependence of K_V_ currents to Equation 1. Fitzmann.m processed *G*–*V* data points and output parameters found in Figure 1, Figure 3, Figure 7, Figure 1—figure supplement 1, Figure 3—figure supplement 2, Table 1, Table 4, and Supplementary file 1a.
