# Supplementary material for: The potassium channel subunit KV1.8 (Kcna10) is essential for the distinctive outwardly rectifying conductances of type I and II vestibular hair cells
Source: eLife. 2024 Dec 3;13:RP94342. doi: 10.7554/eLife.94342 (PMC11614384; doi:10.7554/eLife.94342)
Supplement: Supplementary file 1. — (a) Test of sex differences in hair cell KV channel data. (b) We did not detect a genotype effect on soma size of type I hair cells (HCs). (c) IKir and IH­ were greater in the extrastriola (ES) than striola (S), but did not vary by genotype. [file elife-94342-supp1.docx]

| **Supplemental File 1a**. *Test of sex differences in hair cell K_V_ channel data.* | | | | |  |
| --- | --- | --- | --- | --- | --- |
| Cell Type | Parameter | Subgroup | Male vs Female posthoc p-value | Test | |
| Type I HC | Tail V_half_ | +/+,+/–  –/– | 0.0023 ** ^a^  0.57 | Normal, homogeneous variance  ANOVA: Genotype (2 levels), Sex (2 levels), Zone (2 levels), Genotype*Sex. | |
|  | Tail S | +/+,+/–  –/– | 0.98  0.58 | ANOVA: Genotype (2 levels), Sex (2 levels), Zone (2 levels), Genotype*Sex. Normal, homogeneous variance | |
|  | Tail g_Density_ | +/+,+/–  –/– | 0.999  0.936 | Normal, nonhomogeneous variance  Welch ANOVA: Genotype*Sex | |
| Type II HC | Peak V_half_ | +/+,+/–  –/– | 0.95  0.28 | Normal, homogeneous variance  ANOVA: Genotype (2 levels), Sex (2 levels), Zone (2 levels), Genotype*Sex. | |
|  | Peak S | +/+,+/–  –/– | 0.999  0.97 | Normal, homogeneous variance  ANOVA: Genotype (2 levels), Sex (2 levels), Zone (2 levels), Genotype*Sex. | |
|  | Peak g_Density_ | +/+,+/–  –/– | 0.64  0.43 | Normal, nonhomogeneous variance  Welch ANOVA: Genotype*Sex | |
|  | % inactivation at 30 mV | +/+,+/–  –/– | 0.98  0.82 | Normal, homogeneous variance  ANOVA: Genotype (2 levels), Sex (2 levels), Zone (2 levels), Genotype*Sex. | |

*^a^* g, 0.9. Male K_V_1.8^+/+,+/–^, –85 ± 1 mV (40) vs. Female K_V_1.8^+/+,+/–^, –79 ± 2 mV (12)

| **Supplemental File 1b**. *We did not detect a genotype effect on* s*oma size of type I HCs. The perimeter of cross-sections of basolateral cell bodies was manually measured from immunohistochemistry sections with sufficient autofluorescence in the 488 channel to distinguish cell morphology, and anti-calretinin to label type II hair cells and calyx-only afferents. Cells were measured from two female littermates.* | | | | | |
| --- | --- | --- | --- | --- | --- |
| Cell Type | *Kcna10* Genotype | Age | Weight (g) | Perimeter (μm) | Test |
| Type I HC | +/+  –/– | P117  P117 | 24.9  25.2 | 47 ± 1 (19)  46 ± 1 (28) | ANOVA: p=0.1, power = 0.07 |

| **Supplemental Table 1c**. *Detected zonal but not genotype differences in hair cell I_Kir_ and I_H­_.* | | | | | |
| --- | --- | --- | --- | --- | --- |
| Cell Type | Zone | I_H_ + I_Kir_ current density (-1*pA/pF) | K_v_1.8^+/+,+/–^ *vs* K_v_1.8^–/–^ p-value | ES *vs* Striola p-value | Test |
| Type I HC | ES  Striola | 48 ± 3 (78)  13.0 ± 2 (19) | 0.3 | 4E-9 **** *^a^* | Non-normal, KWA |
| Type II HC | ES  Striola | 55 ± 2 (116)  39 ± 4 (20) | 0.19 (0.25 power) | 0.0058 ** *^b^* | Normal, homogeneous variance. 2-way ANOVA: Genotype (2 levels), Zone (2 levels) |

*^a^* g 1.4

*^b^* g 0.6
